# Systematic investigation of double emulsion dewetting dynamics for the robust production of giant unilamellar vesicles

**DOI:** 10.1039/d6lc00098c

**Published:** 2026-04-22

**Authors:** Wenyang Jing, Heewon Noh, Timothy J. C. Tan, Nicholas C. Wu, Hee-Sun Han

**Affiliations:** a Center for Biophysics and Quantitative Biology, University of Illinois Urbana-Champaign 600 S Mathews Ave Urbana Illinois 61801 USA hshan@illinois.edu; b Department of Chemistry, University of Illinois Urbana-Champaign 505 South Mathews Ave. Urbana Illinois 61801 USA; c Department of Chemical and Biomolecular Engineering, University of Illinois Urbana-Champaign 600 S Mathews Ave. Urbana Illinois 61801 USA; d Department of Biochemistry, University of Illinois Urbana-Champaign Urbana IL 61801 USA; e Carl R. Woese Institute for Genomic Biology, University of Illinois Urbana-Champaign 1206 W Gregory Dr Urbana IL 61801 USA; f Carle Illinois College of Medicine, University of Illinois Urbana-Champaign 807 South Wright St. Urbana Illinois 61801 USA

## Abstract

Giant unilamellar vesicles (GUVs) embody biomimetic membranes with compartmentalization and serve as simplified models to better understand complex biochemical and biophysical processes. Recently, double emulsion droplet microfluidics has proven to be a promising platform for their production, offering greater throughput, control, and reproducibility over traditional methods. However, the interplay of parameters that influence the complex multiphase fluid dynamics of the dewetting process has not been thoroughly studied, limiting the democratization of the approach. In this study, we systematically investigate how lipid composition, aqueous phase conditions, droplet confinement, and fluid dynamics promote or impede dewetting. We reveal that successful GUV formation depends on a critical balance between dynamic Marangoni stresses and thermodynamic interfacial forces under confinement. High surfactant concentrations amplify Marangoni flows and necessitate glycerol for vesicle stability. Conversely, reducing surfactant levels minimizes this dynamic barrier to enable rapid on-chip dewetting, yet imposes thermodynamic constraints that are overcome by tuning lipid conditions. Crucially, in the presence of physiological salt, we identify lipid adhesion energy as the governing parameter; increasing membrane packing *via* saturation successfully overcomes salt-induced inhibition. Our results improve the reliability and accessibility of droplet-microfluidics GUV platforms to catalyze advances in biophysics, synthetic biology, and drug discovery.

## Abstract 2

Decoding dewetting dynamics in double emulsions establishes predictive rules for robust, tunable GUV assembly. This physics-based framework provides systematic guidance to optimize GUV production across varied lipid, aqueous, and fluidic parameters.

## Graphical abstract

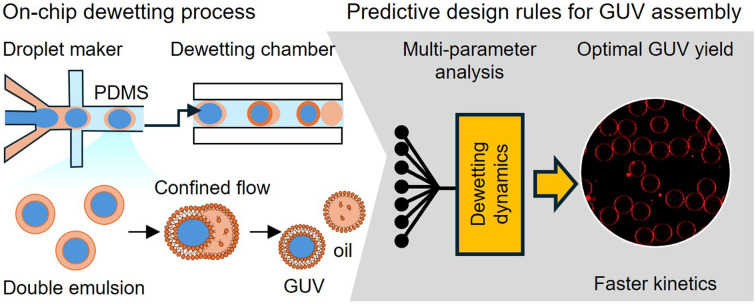

## 1. Introduction

The study of cellular functions is often complicated by the complexity of signaling networks and regulatory circuits, making it challenging to dissect basic mechanisms or evaluate drug efficacy. Giant unilamellar vesicles (GUVs)^1–5^ provide an attractive platform for biochemical and biophysical studies due to their biomimetic lipid bilayer and compartmentalization. Traditional bulk production methods suffer from poor encapsulation efficiency and reproducibility,^2^ whereas droplet microfluidics offers precise control over size, cargo loading, production throughput, and the fluid environment.^6–9^ Among microfluidic strategies, the double emulsion (DE)-templated method is widely adopted for high-throughput GUV production.^9–17^ In this method, a lipid-laden oil shell separates the inner (IA) and outer aqueous (OA) phases; this shell spontaneously dewets to form a stable bilayer, mimicking cytosolic and extracellular compartments. Compared with single emulsion methods^8,18^ that rely on surfactant-stabilized interfaces and droplet break-up, the DE approach is gentler, yielding superior throughput and monodispersity.

Despite their promise, DE-templated GUVs are largely constrained by a reliance on fixed, empirical recipes. Because the underlying physics of dewetting and vesicle formation is not well understood, researchers must rely on a narrow set of chemical conditions known to work, typically involving high concentrations of additives like poloxamer 188 (P188) and glycerol.^9–13,17^ This rigidity limits the method's versatility in two critical ways. First, these additives fundamentally alter the physical properties of the lipid membranes,^19–23^ complicating biophysical studies of membrane mechanics.^24–26^ Second, such a chemical environment can be incompatible with sensitive biological systems, affecting protein function or interfering with other functional assays.^9,15,19,27–31^ Simply reducing glycerol to a more biocompatible regime^15,27–29^ is often unfeasible, as GUVs become unstable and rapidly disintegrate even at lower P188 concentrations. While eliminating P188 has been achieved by increasing lipid concentration,^14^ this method is slow, requiring minutes to hours for formation and still relies on additives like glycerol to ensure stability. Moreover, attempts to move beyond standard experimental conditions are largely matters of trial-and-error, as improving one parameter often compromises another. Together, these limitations highlight the central problem: without understanding the phase diagram, the field remains trapped in a local optimum.

Dewetting of double emulsions is a complex self-assembly process governed by interacting physicochemical and fluid dynamic parameters. Previous theoretical frameworks have focused primarily on thermodynamics, neglecting the dynamic contributions that we find to be critical: flow-induced Marangoni stresses can, in some conditions, reverse trends predicted by thermodynamics alone. This complex interplay of thermodynamics and kinetics, together with the dependence of throughput on dewetting rates and the need to tune lipid and aqueous compositions for specific applications, explains why robust design guidelines have been lacking and why DE GUV production has relied largely on trial-and-error. To our knowledge, no study has systematically investigated this multidimensional parameter space, highlighting the critical need for mechanistically-grounded design principles.

Here we present the first systematic investigation of multiphase DE dewetting, providing design rules to transform DE-templated GUV production into a flexible, predictable platform. We systematically varied a wide range of properties, including lipid concentration,^14^ charge, acyl chain saturation, surfactant levels, aqueous solution composition,^32^ on-chip flow speed, and confinement,^33^ to map the phase diagram of GUV formation. We found that dewetting behavior is non-monotonic and context-dependent; static thermodynamic models alone cannot predict outcomes.

The primary contribution of this work is the establishment of mechanistically grounded design principles that replace empirical guesswork with predictive guidance. This framework enables access to previously challenging experimental regimes. We demonstrate this utility by identifying the precise conditions required to eliminate glycerol and reduce P188 to 0.1%, the standard in cell cultures,^34^ resolving the long-standing toxicity issues. Furthermore, our design rules allow GUV formation at physiological salt concentrations; although salt impedes dewetting, tuning adhesion energy *via* lipid saturation overcomes this barrier. In addition, our streamlined microfluidic design integrates DE production and on-chip dewetting, enabling high-throughput GUV generation at approximately 600 Hz, stably maintained for several hours, and seamlessly integrating with a broad range of downstream assays. Our study establishes a robust foundation for tuning GUV production, extending its applicability beyond narrow assay conditions to diverse biophysical characterization and synthetic cell engineering applications.

## 2. Materials and methods

### Reagents

1,2-Dioleoyl-*sn-glycero*-3-phosphocholine (DOPC), 1,2-dioleoyl-*sn-glycero*-3-phospho-rac-(1-glycerol) sodium salt (DOPG), 1-palmitoyl-2-oleoyl-*glycero*-3-phosphocholine (POPC), 1-palmitoyl-2-oleoyl-*sn-glycero*-3-phospho-(1′-rac-glycerol) sodium salt (POPG), 1,2-dipalmitoyl-*sn-glycero*-3-phosphocholine (DPPC), and 1,2-dioleoyl-*sn-glycero*-3-phosphoethanolamine-*N*-(lissamine-rhodamine-B-sulfonyl) ammonium salt (Liss Rhod PE) were purchased from Avanti Research. *N*-(7-Nitrobenz-2-oxa-1,3-diazol-4-yl)-1,2-dihexadecanoyl-*sn-glycero*-3-phosphoethanolamine triethylammonium salt (NBD PE), cholesterol (purity ≥99%), 1-octanol (purity ≥99%), pluronic F-68 (P188), dextrose, sucrose, sodium chloride, potassium chloride, poly(diallyldimethylammonium chloride) (PDADMAC), poly(sodium 4-styrenesulfonate) solution (PSS), and poly(vinylalcohol) (PVA, Mw 13 000–23 000, 87–89% hydrolyzed) were purchased from Sigma Aldrich. HEPES (1 M, corning) was purchased from VWR.

### Device fabrication

The double emulsion generating chip was fabricated using the standard methods of photolithography, utilizing two junctions with a short gap of 15 μm to promote stable jetting of the IA and oil phases while avoiding Plateau–Rayleigh instability (diagram in Fig. S1A), thus enabling double emulsion formation at the second junction without consideration of droplet generation frequency at the first junction. The microfluidic device was designed based on the prior work.^9^ The microchannel was fabricated using conventional soft lithography (described below). Channel features were measured using a Dektak profilometer, with the droplet making channel height at approximately 13 μm and junction widths of 10 μm. We made approximately 16 μm double emulsions against the dewetting channel heights of 13.5, 20, and 30 μm to change confinement, with varying widths to achieve different flow speeds at each confinement.

Polydimethylsiloxane (PDMS) was poured onto the silicon wafer at a mass ratio of 10 : 1. After curing, PDMS layer was peeled off following overnight baking at 65 °C and punched with a biopsy punch. Subsequently, the PDMS layer, another PDMS slab, and a cleaned glass slide were exposed to a plasma cleaner for 15 s and then bonded in the following order: PDMS layer, the flat PDMS slab, the glass slide. The assembled device was further baked overnight at 65 °C to ensure full recovery of a hydrophobic PDMS surface.

### Surface treatment

For the droplet generator, the first junction and gap to the second junction requires a hydrophobic surface while the second junction and the region going to the outlet require a hydrophilic surface. To achieve this, we utilized strong polyelectrolytes, namely 5% PDADMAC and 5% PSS, which are positive and negative in solutions, respectively. We utilized plasma treatment to layer PDADMAC, providing a safer and more efficient alternative to piranha-based or hydrogen peroxide solutions, which produce significant gas. A fully assembled chip has the outlet exposed and the other punched holes covered in tape and is exposed to plasma in a similar manner during bonding. The time will vary depending on channel geometry and distance of junction from the outlet. Under-exposure will prevent adequate surface treatment, but slight over-exposure is acceptable as water is used to confine the flow^35^ of the polyelectrolyte solution to the second junction (see Fig. S2) and the post-treatment baking restores hydrophobicity to the untreated PDMS surface. Specifically, we plug the oil inlet, flow water from the IA inlet, and polyelectrolyte from the outlet. After the interface forms, we wait for 5 min while the water and polyelectrolyte flow out of the OA inlet. After the PDADMAC treatment, it is disconnected from the outlet and water will continue to flush the channel for another 5 min before introducing the PSS solution. It is important to allow for enough flushing in between or the mixing of PDADMAC and PSS in solution will aggregate and clog the channel. The device was then baked at 65 °C overnight to heat-immobilize the PDADMAC/PSS layers onto the device and restore the hydrophobic surface of the untreated portion. This process allowed for the reusability of the device, enabling the generation of w/o/w droplets at least twice.

The dewetting chips are PDMS bonded to glass and treated with 5% of PVA by allowing it to incubate for several minutes before blowing it out with nitrogen and then baking at 120 °C for 15 min. However, we find this to not be deterministic for whether dewetting occurs on-chip as untreated and treated dewetting chips show similar outcomes for all parameters tested.

### Preparation of oil and aqueous phase solutions

Pure lipid stocks were purchased from Avanti Polar Lipids, Inc., and stored in chloroform at −20 °C. Cholesterol was purchased in solid form from Sigma-Aldrich. LO solutions were prepared from these stocks in chloroform, the compositions of which are detailed in other sections. To visualize GUVs, the fluorescent Liss Rhod PE (0.1%) or NBD PE (0.5%) was added, as a fully dewetted GUV has poor contrast under brightfield as opposed to a thin-shell double emulsion. Chloroform evaporated under a gentle stream of argon, and then lipid films were dried under vacuum for at least 2 h. Then 1-octanol was added to achieve a final concentration of 5–35 mg mL^−1^.

All IA and OA solutions were titrated to pH 7.6 before use. The IA solutions were: 0.5 M sucrose and 0.02% P188 (Fig. 3A–C and 4A–C); 10 mM HEPES, 0.1 M NaCl, 0.29 M sucrose, and 0.02% P188 (Fig. 3D–F and 4D–F); and 10 mM HEPES, 0.1 M KCl, 0.29 M sucrose, and 0.02% P188 (Fig. 8). The OA solutions were: 0.5 M glucose and 5% P188 (Fig. 3A–C); 10 mM HEPES, 0.1 M NaCl, 0.29 M glucose, and 5% P188 (Fig. 3D–F); 0.5 M glucose and 0.1% P188 (Fig. 4A–C); 10 mM HEPES, 0.1 M NaCl, 0.29 M glucose, and 0.1% P188 (Fig. 4D–F); 10 mM HEPES, 0.1 M KCl, 0.29 M glucose, and 0.1% P188 (Fig. 8). Sucrose and glucose were added to the IA and OA, respectively, to match the osmotic balance during on-chip dewetting and create a density difference.

### Microfluidic device operation

We employed a two-stage system^11,12^ comprising a separate droplet maker and dewetting chamber connected by PE tubing. In this setup, DE droplets are generated in the first chip and undergo phase separation in the second. This two-stage configuration offers several advantages over conventional single-stage chips.^9^ First, it enables straightforward, single-layer fabrication of each component with distinct heights, eliminating the need for aligning different PDMS layers to create a multi-height device. Second, it allows for separate surface treatments of the droplet maker and dewetting chamber, streamlining fabrication and significantly increasing the success rate. Producing stable DEs requires a sharp transition from a hydrophobic to a hydrophilic surface at the droplet-making junction. In a single-stage system, selectively depositing the hydrophilic polymer at this junction is challenging because it is typically introduced from the outlet of a long, wide dewetting chamber. Our two-stage design isolates the droplet maker, enabling a simple and precise plasma treatment-based polymer deposition that yields more reliable DE production.

Our two-stage setup, combined with the use of biocompatible, glycerol-free conditions, improves GUV production throughput by orders of magnitude. Since the dimensional and speed requirements for high-throughput droplet generation and efficient dewetting differ, separating the droplet generation and dewetting processes allows for independent optimization of each step. Furthermore, eliminating glycerol and reducing the P188 concentration lowers the capillary number (Ca = *ηU*/*γ*, where *η* is the dynamic viscosity, *U* is flow speed, and *γ* is the interfacial tension). This reduction can suppress jetting at the droplet-making junction, which is a critical factor for achieving small channel dimensions required to produce 10–20 μm GUVs.

Previous study^9^ operated at very low IA flow rates (∼1 μL h^−1^), which are impractical for standard syringe pumps and can lead to channel clogging over time due to octanol buildup.^36^ In contrast, we operated our droplet maker at significantly higher and more stable flow rates (IA/LO/OA: 15/15/40 μL h) using a standard syringe pump (KDS Milliliter, OEM pump), though this is not the upper limit. For a typical experiment, we prepared 300 μL of solution and utilized approximately 10 μL per run. The maximum on-chip residence time for dewetting in the second chip was ∼30 s, an order of magnitude lower than previously reported^9^ and sufficient for our systematic investigation.

Within the dewetting chamber, we systematically modulated the key physical parameters. Flow velocity and channel height were modulated by altering the dimensions of the dewetting chamber with fixed flow rates. Compared to the original study^9^ (∼0.5 mm s^−1^), three average flow velocities were tested and spanned an order of magnitude from ∼1 mm s^−1^ to ∼20 mm s^−1^, with the corresponding Reynolds numbers (Re) ranging from ∼0.03 to ∼0.5, where Re = *ρUL*/*η*, *ρ* is the fluid density, *U* is flow speed, *L* is the hydraulic diameter of the channel, and *η* is the dynamic viscosity. Additionally, we examined three different confinements—0.5, 0.8, and 1.2—defined as *a*/*H*, where *a* is the droplet diameter and *H* is the height of the dewetting channel.

A specific modification was required for experiments using 5% P188 in the outer aqueous (OA) phase, as this composition failed to form droplets at the junction. To address this, we added an inlet downstream of the droplet-maker to introduce and homogenize additional P188 with the DEs before entering the dewetting chamber (Fig. S3).

### Visualization of on-chip dewetting process and outcome

On-chip formation and dewetting of double emulsion droplets were recorded using a Zeiss Axio Observer 5 inverted microscope equipped with a Photron MiniAX200 high-speed camera. Wide-field and fluorescence images of collected samples were acquired at room temperature using the same system, with excitation *via* BP 470/40 (GFP) and BP 560/40 (Texas Red) filter sets.

High-resolution confocal fluorescence and bright-field images were acquired with a confocal laser scanning microscope (LSM 880 inverted confocal laser scanning microscope, Zeiss). For fluorescence visualization, samples were excited using the 488 nm (Argon) and 561 nm (DPSS) laser lines. Transmitted light images were captured using a transmitted light PMT (T-PMT) for bright-field imaging. All images were acquired using 40×/1.2 water immersion objective and processed using ZEN lite (version 3.8) software.

### α-Hemolysin membrane leakage assay

To functionally validate the produced liposomes, we reconstituted α-hemolysin (α-HL), a pore forming protein, into liposomes and monitored leakage of encapsulated small molecules, as described in the original study.^9^ Time-series confocal images were acquired using 488 nm (argon) and 561 nm (DPSS) laser lines following the addition of 10 μg mL^−1^ α-HL to dewetted GUVs encapsulating 10 μM calcein. Control experiments were performed under identical conditions in the absence of α-HL.

### Centrifugation

Centrifugation, an external high-speed flow system, is applied to some conditions to further assist the dewetting^15^ as octanol (0.82 g mL^−1^) is less dense than the aqueous phase (1.04 g mL^−1^). To assess the centrifugation-driven dewetting dynamics, partially dewetted droplets collected from the dewetting chamber were subjected to benchtop centrifugation at three different forces: 2000 × *g* for 1 min, 10 000 × *g* for 1 min, and 17 000 × *g* for 1 min. While increasing the centrifugal force or the duration should enhance the separation, our observations revealed that excessively high mechanical stresses caused GUV fragmentation (Fig. S4), consistent with a prior study.^37^ We carefully optimized the centrifugation conditions to balance effective dewetting with minimal GUV fragmentation. By contrast, direct centrifugation of DE droplets resulted in prolonged dewetting, which is undesirable due to the increased risk of GUV fragmentation during extended centrifugation.

### Surface tension measurements

The interfacial tensions between IA and LO and that between OA and LO were measured at the Materials Research Laboratory Central Research Facilities, University of Illinois, using pendant drop method on a Ramé-Hart model 250 contact angle goniometer. In this setup, droplets of each aqueous solution were placed in oil, and the contact angles between the aqueous drop and the surrounding oil were measured. The compositions are detailed in Fig. S5 and S6.

### Cell culture

HEK293T landing pad (HEK293TLP) cells,^38^ a kind gift from Kenneth Matreyek (Case Western Reserve University), were grown and maintained in D10 + Dox medium: Dulbecco's modified Eagle medium (DMEM; Gibco) with 10% v/v fetal bovine serum (FBS, Gibco), 1× GlutaMAX (Gibco), 100 U mL^−1^ penicillin and 100 μg mL^−1^ streptomycin (Gibco), and 2 μg mL^−1^ doxycycline (Thermo Fisher Scientific). HEK293TLP cells stably expressing on its surface SARS-CoV-2 spike with the “PRRA” motif in the furin cleavage site deleted and an A944S mutation, and intracellularly expressing mNG2_11_ fused to the C-terminus of GCN4 (HEK293TLP-SΔPRRA-A944S-mNG2_11_) were constructed previously^39^ and grown in puromycin medium – D10 + Dox medium supplemented with 1 μg mL^−1^ puromycin (Invivogen). HEK293TLP cells stably expressing on its surface human ACE2 (HEK293TLP-hACE2) were constructed previously^39^ and grown in hygromycin medium – D10 + Dox medium supplemented with 100 μg mL^−1^ hygromycin B gold (Invivogen). HEK293TLP-hACE2 cells intracellularly expressing mNG2_1–10_ fused to the C-terminus of GCN4 (HEK293TLP-hACE2-mNG2_1–10_) were constructed previously^39^ and grown in hygromycin medium. All cells were incubated at 37 °C, 5% CO_2_, and 95% humidity.

### Co-culture assay

2.5 × 10^5^ HEK293TLP-SΔPRRA-A944S-mNG2_11_ cells were mixed with an equal number of HEK293TLP-hACE2-mNG2_1–10_ cells in a total of 2 mL of D10 + Dox medium in wells of a 6-well plate. Pluronic F-68 was added to co-cultures achieve final concentrations of 0%, 0.1% and 1% w/v. For non-fluorescent control, HEK293TLP-SΔPRRA-A944S-mNG2_11_ cells were mixed with HEK293TLP-hACE2 cells without P188 added. Co-cultures were incubated 37 °C, 5% CO_2_, and 95% humidity for 24 h.

### Flow cytometry

Twenty-four hours post-co-culture, cell supernatant for cells co-cultured in 1% pluronic F-68 was harvested. For all other conditions, cells were washed once with 1× PBS, trypsinized, and then harvested. Cell suspensions were centrifuged for 300 × *g* at 4 °C for 5 min, and the supernatant was discarded. Cell pellets were resuspended in ice-cold flow cytometry buffer: DMEM without phenol red (Gibco) supplemented with 2% v/v FBS and 5 mM EDTA. Then, cells were analyzed using an Accuri C6 flow cytometry (BD Biosciences), and data were acquired using Accuri C6 software (BD Biosciences). Flow cytometry data were analyzed with FCS Express v6 (*De Novo* Software). Gating strategy is shown in Fig. S5.

## 3. Thermodynamics *vs.* kinetics in dewetting

**Fig. 1:**
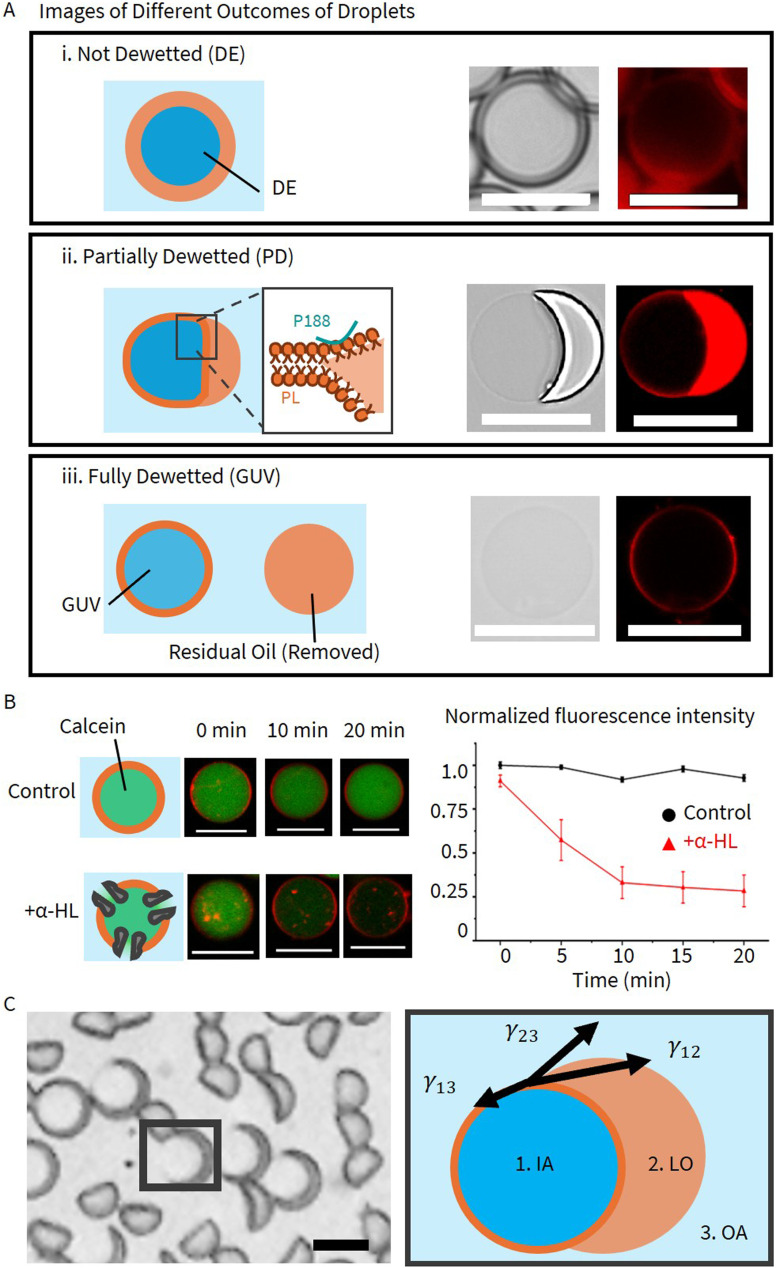
(A) Three outcomes of dewetting: (i) double emulsions with oil shells, imaged *via* wide-field microscopy; (ii) partially dewetted vesicles with side-attached oil pockets, and (iii) fully dewetted GUVs, both imaged *via* confocal microscopy. Panel shows an illustrative depiction (left) and brightfield and fluorescence images (right). GUVs are labeled with 0.2% Liss Rhod PE, a fluorescent lipid dye. P188 refers to pluronic F68, and PL denotes phospholipids. Scale bar: 20 μm. (B) GUV unilamellarity confirmed by α-hemolysin (α-HL) leakage assay. Normalized fluorescence decay for α-HL-treated GUVs shows calcein (green) release from α-HL-treated GUVs (DOPC : DOPG 2 : 1 mol%, 0.1% P188, no glycerol, no salt) upon pore formation. In contrast, control GUVs retain fluorescence intensity over the same period. Error bars represent mean ± SD (*n* = 10). (C) Three interfacial tension values involved in the dewetting of DE droplets. The DE droplet in the highlighted frame is shown as an example. Scale bar: 20 μm.

On-chip DE-templated GUV production relies on the spontaneous phase separation of DE droplets under flow and confinement. In an ideal transition, the oil pocket shifts, and the lipid bilayer zips up, driving the oil to pinch off to form a GUV. In practice, however, we observe three outcomes: non-dewetted DE droplets, partially dewetted (PD) states where the oil remains attached, and fully dewetted GUVs (Fig. 1A). Conventionally, this process is described by surface energy minimization and the spreading coefficient,^40–42^ which depends on the three interfacial tensions involved (Fig. 1C, SI). However, this static thermodynamic framework assumes a stationary droplet at equilibrium and ignores the dynamic reality of on-chip operation, where droplets experience continuous shear and deformation.

Our experiments highlight the strong influence of dynamic factors such as confinement and flow rate. These factors are known to shape the dewetting pathway, timescale, and even the final state *via* dynamic viscosity changes^43^ and fluid flow.^44–46^ Simulations show that high confinement reorganizes lipids and alters membrane tension gradients.^47^ Shear flow, which is coupled to confinement, also creates interfacial tension gradients that produce Marangoni stresses^48–50^ that can inhibit dewetting. Even conservatively, we estimate the Marangoni number to be substantial, on the order of ∼10–100 (SI), reinforcing that thermodynamic considerations alone are insufficient to describe dewetting dynamics. As a result, dewetting in this system is generally metastable and path-dependent, underscoring the need for systematic experimental characterization. To build practical, predictive guidance beyond static theory, we systematically examined how the dewetting process responds to variations in P188 concentration, lipid charge, lipid concentration, salt and buffer composition, confinement, flow velocity, and lipid acyl chain saturation.

## 4. Results and discussion

### 4.1 Classification of dewetting dynamics

**Fig. 2:**
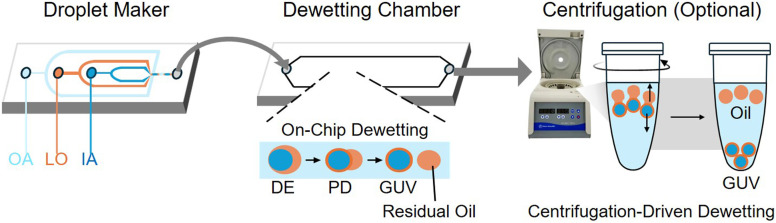
Experimental setup for investigating GUV dewetting dynamics. A two-stage microfluidic system is used to first generate double-emulsion (DE) droplets and then induce dewetting under controlled flow conditions. For cases of partial dewetting, a secondary centrifugation step is employed to drive the process to completion. DE and PD denote double emulsion and partially dewetted, respectively.

Under optimized conditions, the device operates at a generation frequency of ∼600 Hz and remains stable for several hours, generating a couple of million GUVs per hour. To quantify the outcomes of our multi-parameter screen, we expanded the standard binary classification of success *versus* failure into a detailed kinetic scheme based on the location and timescale of the process (Fig. 2). Outcomes were categorized as “on-chip”, defined as complete dewetting within the device, which occurs within a maximum of ∼30 s; “off-chip”, denoting complete dewetting occurring within hours of collection in a collection tube; or “partial dewetting”, characterized by incomplete separation where residual octanol remained attached even after off chip incubation. For “on-chip” cases, dewetting time was calculated using the average flow speed and the linear distance required for the entire DE population to transition to GUVs. As this metric is derived from the spatial transition point of the continuous droplet stream, it represents the collective behavior of hundreds of droplets rather than isolated tracking events. The associated uncertainty stems from visual variability in identifying the precise transition point, minor pump pulsations, and slight variations in channel height. For partial dewetting cases, we centrifuged the drops to assess whether the remaining kinetic barrier could be overcome by centrifugal shearing. Detailed protocols are provided in the materials and methods section.

### 4.2 Unveiling high surfactant dewetting mechanisms

To isolate the underlying kinetics of dewetting, we first investigated the process at a high surfactant concentration (5% P188). Consistent with previous protocols,^9,10,15,17^ we confirmed that glycerol is essential for forming intact GUVs in this regime (Fig. S6). Eliminating glycerol results in a consistent failure mode: vesicle rupture,^32,51^ which yielded free-standing octanol droplets and a high fluorescence background (Fig. S7). This result indicates that the forming vesicle cannot fully dewet while maintaining its integrity, allowing the inner and outer phases to merge. We attribute the stabilizing effect of glycerol to its ability to increase solvent and membrane viscosity,^19^ which dampens destructive tension gradients. Although this glycerol-free process does not yield intact GUVs, the dewetting and rupture occurs over a measurable timescale of approximately 30 seconds, providing a valuable window to systematically study the underlying kinetics of the process.

**Fig. 3:**
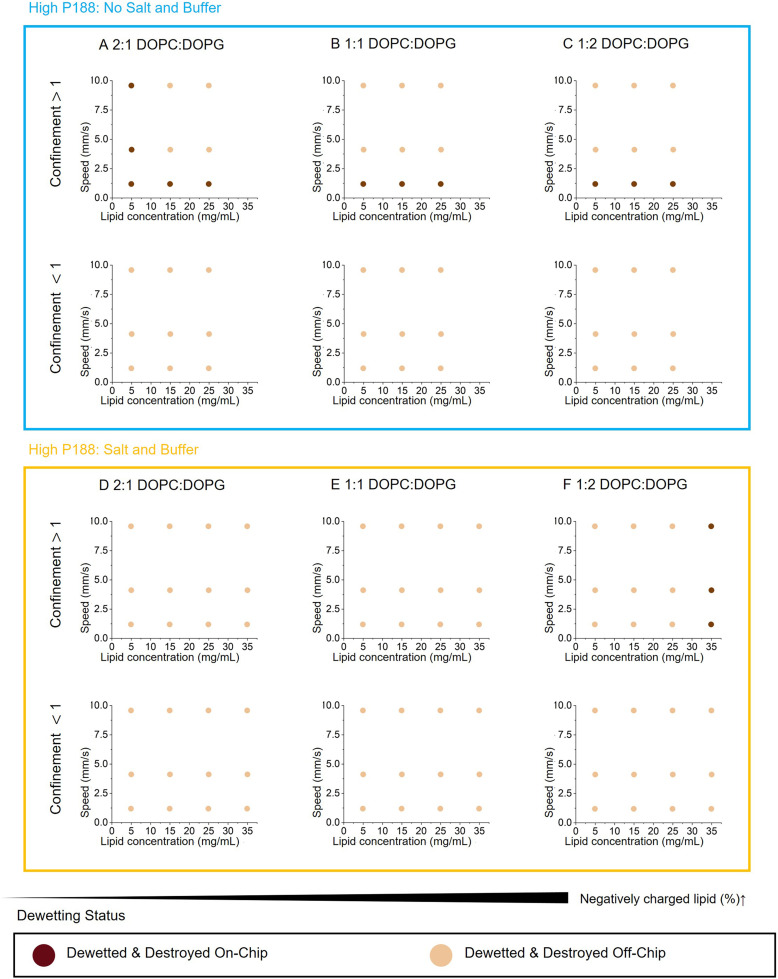
Dewetting results at high OA surfactant concentration (5% P188) without glycerol. These plots show the results of double emulsion dewetting for different negative charge ratios, lipid concentrations, on-chip flow speeds in the dewetting chamber, confinement during dewetting, and the presence or absence of salt and buffer in the OA (see materials and methods). A and D utilize 2 : 1 DOPC : DOPG, B and E utilize 1 : 1 DOPC : DOPG, and C and F utilize 1 : 2 DOPC : DOPG. A–C lack salt or buffer in aqueous phase while D–F include 10 mM HEPES, 0.1 M NaCl, 0.29 M sucrose (IA) or glucose (OA). We note that dewetting universally occurred, however no meaningful amount of GUVs survived the process intact.

In the 5% P188 regime, the dewetting and rupture eventually occurred across all conditions, confirming that the merging of IA and OA phases is thermodynamically favorable, as detailed in the SI. However, while the dewetting itself was rapid once the oil pocket formed, the induction time prior to onset varied significantly among individual droplets. This indicates that the process is stalled by kinetic barriers. We therefore analyzed the total population collectively and classified outcomes as either “dewetted & destroyed on-chip” or “dewetted & destroyed off-chip” (Fig. 3) to elucidate kinetic factors. Notably, even among samples classified as ‘dewetted and destroyed on-chip,’ the specific spatial location of these events varied across conditions, further aligning with the observed kinetic trends.

We identified three distinct kinetic drivers: confinement, Marangoni stresses, and ionic environment. High confinement, defined as a channel height smaller than the droplet diameter, was required for on-chip dewetting. This finding suggests that confinement enhances internal circulation through droplet tank-treading, consistent with a previous study showing that internal circulation speeds up the transition from the completely engulfed or core-shell state to the partially dewetted state.^52^ Therefore, all analyses of subsequent dynamics were conducted under high confinement.

In salt-free conditions, we observed a counter-intuitive trend where faster flow speeds and higher lipid concentrations impeded on-chip dewetting (Fig. 3A–C). This finding contradicts earlier reports suggesting that higher shear stress enhances dewetting^14,33^ and that increasing lipid concentration promotes dewetting *via* depletion forces.^14,53^ Instead, our results point to a dominant role of the Marangoni effect. High lipid and P188 concentrations, coupled with strong shear, induce significant interfacial tension gradients.^43,47–50^ These gradients generate Marangoni stresses that can oppose dewetting forces and compromise membrane integrity, hindering the kinetics of the thermodynamically favorable transition.

This mechanism was further supported by the influence of lipid charge, a previously unexplored variable in this context but noted in other system.^54^ Increasing negative charge decreases both the inner and outer interfacial tensions (SI), similar to the effect of increasing lipid concentration, and would be expected to promote dewetting. Yet we observed the opposite: more negatively charged compositions dewetted more slowly. Moreover, lipid charge introduced a flow-dependent failure mode: at higher charge ratios (1 : 2 and 1 : 1 DOPC/DOPG), faster flow suppressed dewetting, whereas the lowest negative charge (2 : 1 DOPC/DOPG) at the lowest lipid concentration dewetted on-chip across all flow speeds (Fig. 3C). This flow-dependent suppression confirms that increased lipid charge amplifies the Marangoni effect in the absence of salt, creating a dynamic barrier to thermodynamic driving force.

Introducing physiological buffer (Fig. 3D–F) reversed the trends. In contrast to salt-free conditions, the addition of salt mitigated this inhibitory flow effect, shifting the system into regime where high lipid or charge concentrations became necessary to drive dewetting. Only the highest concentration (35 mg mL^−1^) and highest charge (1 : 2 DOPC/DOPG) dewetted on chip (Fig. 3D). We attribute this reversal to the ability of salt to dampen lipid mobility,^55,56^ increasing the viscosity of the aqueous phase, and enhancing membrane rigidity and thickness through strong electrostatic interactions with lipid head groups, which diminishes the effect of Marangoni stress. These behaviors, which arise from the interplay of lipid composition, salt, and flow, cannot be predicted by static thermodynamic models with the spreading coefficient. Instead, they highlight the intrinsically kinetic and path-dependent nature of dewetting dynamics.

### 4.3 Achieving rapid GUV production: low surfactant dynamics

**Fig. 4:**
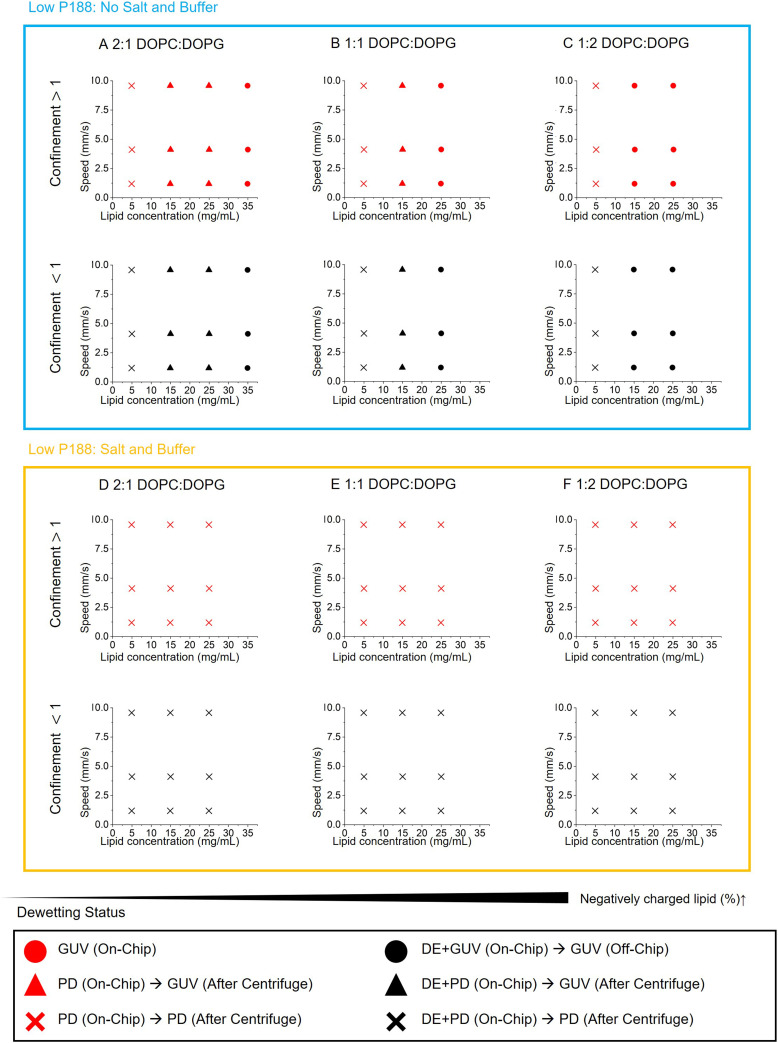
Dewetting results at low OA surfactant concentration (0.1% P188). Similar to Fig. 4, A–C are for no salt or buffer in the aqueous while D–F contains 10 mM HEPES, 0.1 M NaCl, 0.29 M sucrose (IA) or glucose (OA). The four on-chip dewetting statuses are categorized as follows: 1) DE dewetted and produced intact GUVs within the dewetting-chip (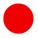), 2) a fraction of DEs dewetted within the maximum on-chip time of ∼30 s but the remainder slowly dewetted off-chip (●), 3) DEs that partially dewetted on-chip (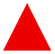) or passively off-chip (▲) could be successfully dewetted after centrifugation, 4) DEs did not dewet even at 17000 × *g* for 1 min (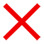 and ×).

In contrast to the destructive rupture observed at 5% P188, lowering the surfactant concentration to 0.1% P188 in the absence of glycerol drastically altered the dynamics, enabling the rapid formation of intact GUVs in seconds. However, this regime introduced a new challenge: the dewetting process sometimes failed to complete on-chip, resulting in partially dewetted states that could persist for several days. This stalling aligns with surface tension measurements (SI), which indicates that the lower P188 concentrations in the OA reduce the driving force. To broaden the operational window, we found that post-collection centrifugation could facilitate complete dewetting under certain conditions. This necessitated a more detailed classification of outcomes for the 0.1% P188 regime (Fig. 4): 1) on-chip full dewetting (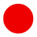), 2) off-chip full dewetting (●), 3) partial dewetting on chip but dewetting completed after centrifugation (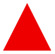 and ▲), and 4) partial dewetting persisting even after centrifugation (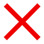 and ×). To confirm that these production routes yield functional unilamellar liposomes despite removing glycerol and reducing surfactant, we performed an α-hemolysin (α-HL) membrane leakage assay (Fig. 1B). The addition of α-HL to dewetted GUVs (DOPC DOPG 2 : 1 mol%, 0.1% p188, no glycerol, no salt) led to significant leakage of encapsulated calcein compared to controls. This result confirms the formation of lipid bilayer and aligns with previous studies of GUVs prepared without P188 and glycerol.^14,57^

The shift to 0.1% P188 fundamentally changed the kinetic landscape. Consistent with 5% P188 regime, confinement greater than 1 was required for on-chip dewetting. However, the dynamics were markedly different. At 0.1% P188, the inhibitory effect of flow speed diminished, and the timescale of dewetting decreased substantially, occurring within the first 10 seconds (Table S1). This result indicates that opposing Marangoni stresses are significantly weaker at this lower concentration. Even in cases of partial dewetting, the transition from DE to the partially dewetted state occurred rapidly under higher confinement (Fig. S8).

With the dynamic Marangoni stresses weakened, the influence of thermodynamics became more apparent in the absence of salt. Increasing lipid concentration and charge acted synergistically to promote dewetting (Fig. 4A–C). At the lowest lipid concentration, 5 mg mL^−1^, dewetting failed entirely, even after days off-chip or after centrifugation, regardless of charge. Conversely, increasing the concentration to 25–35 mg mL^−1^ enabled complete on-chip dewetting, aligning with thermodynamic depletion theories.^14,53^ Furthermore, higher fractions of negative charge promoted dewetting; for example, while a high lipid load of 35 mg mL^−1^ was necessary for low-charge formulations (33% DOPG), moderate loads of 15 mg mL^−1^ were sufficient for high-charge formulation (67% DOPG). This charge dependence may be driven by lower solubility of charged lipids in oil, evidenced by low-temperature precipitation (data not shown). This thermodynamic incompatibility can increase effective adhesion energy to zip the opposing monolayers into the stable bilayer, as observed in DIB experiments (SI). Overall, successful on-chip dewetting under restricted P188 conditions requires a sufficiently high lipid concentration and charge.

**Fig. 5:**
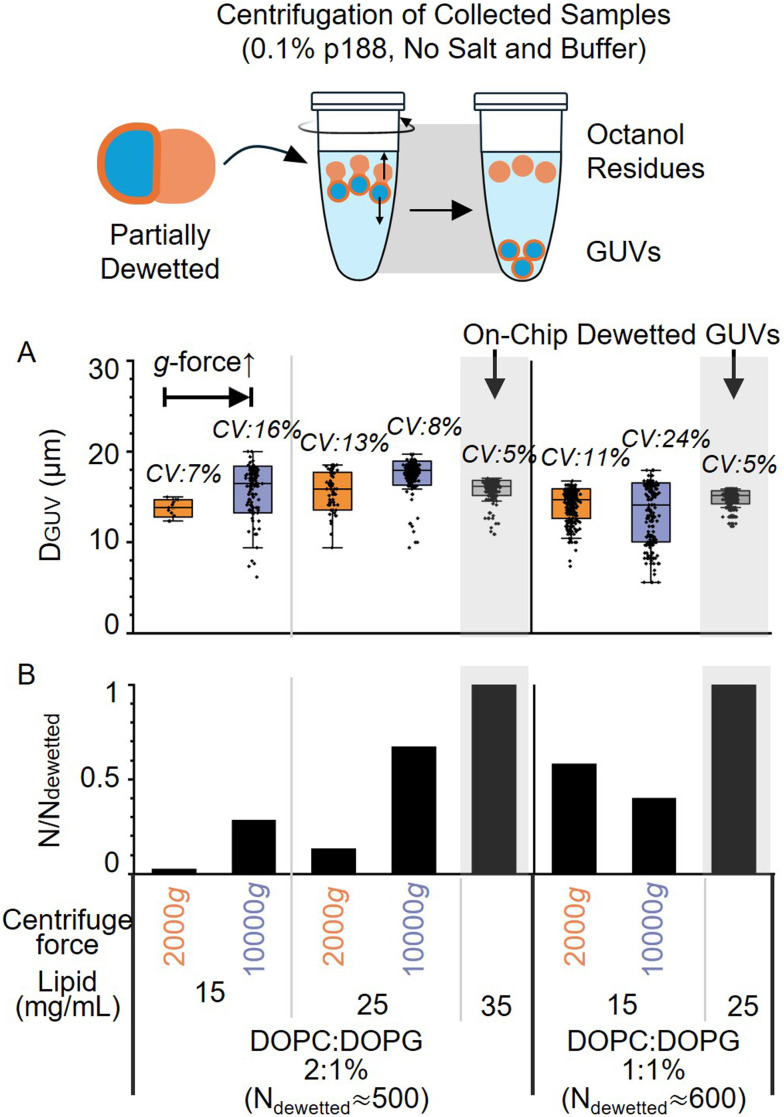
Quantification of centrifugation-driven GUV formation with low P188, no salt. All samples were generated using identical flow rates and collection times. (A) GUV diameter distributions for on-chip (grey background) *versus* centrifugation-driven dewetting. Data are presented as mean ± SD (box boundaries). (B) Relative GUV formation efficiency, defined as the number of centrifugation-dewetted GUVs normalized to on-chip dewetted controls (*N*_dewetted_ ≈ 500–600 GUVs). Values < 1.0 indicate incomplete dewetting, with a portion of vesicles stalled in partially dewetted states. Lipid compositions and specific centrifugation conditions are indicated for each sample.

Lipid charge and concentration exert similar effects during post-chip centrifugation, where increased centrifugal shear flow is applied to overcome the kinetic barrier in stalled droplets: high lipid concentration and charge lower the energy barrier for centrifugation (Fig. 5 and S9). Conditions prone to stalling (5 mg mL^−1^ lipid) presented such a high barrier that they failed to dewet even at maximum force (17 000 × *g*). In contrast, optimized conditions (25 mg mL^−1^ DOPC/DOPG 2 : 1) dewetted effectively at gentle forces (2000 × *g*). This correlation allowed us to tune the lipid composition to minimize the required centrifugal force, thereby avoiding fragmentation. Under these optimized conditions, centrifugation yielded GUV populations with slightly higher size dispersity than spontaneous on-chip formation (coefficient of variation (CV): 8–11% *versus* 5%, respectively), effectively converting the stalled population into high-quality vesicles. The slightly higher polydispersity, however, demonstrates that while both separation methods are governed by the same physical principles, the on-chip process remains the inherently gentler pathway to overcome kinetic barriers.

Despite the success of the low-surfactant regime in salt-free conditions, the inclusion of physiological buffer (10 mM HEPES, 100 mM NaCl) completely arrested the process (Fig. 4D–F). None of the conditions achieved complete dewetting, even with extensive centrifugation (Fig. S10). These findings are consistent with a previous study showing partial dewetting under physiological ionic concentrations, such as 180 mM potassium glutamate, even with 1% P188.^32^ Salt likely impedes the process by increasing interfacial tensions and the viscosity of the aqueous phase and reducing lipid mobility.^55,56^ These effects hinder intermolecular shuffling and the expulsion of the oil, both essential for successful dewetting. Another study on liquid–liquid phase separation of polymeric composite particles supports this observation, showing that kinetic barriers, such as increased viscosity of the liquid phase from solvent evaporation, can prevent the system from reaching its predicted thermodynamic equilibrium.^58^ While our systematic framework successfully resolved the dynamic constraints associated with surfactant reduction and glycerol removal, achieving physiological compatibility requires identifying an additional thermodynamic control parameter to counterbalance the kinetic barrier imposed by salt.

### 4.4 Influence of fluid dynamics on dewetting

**Fig. 6:**
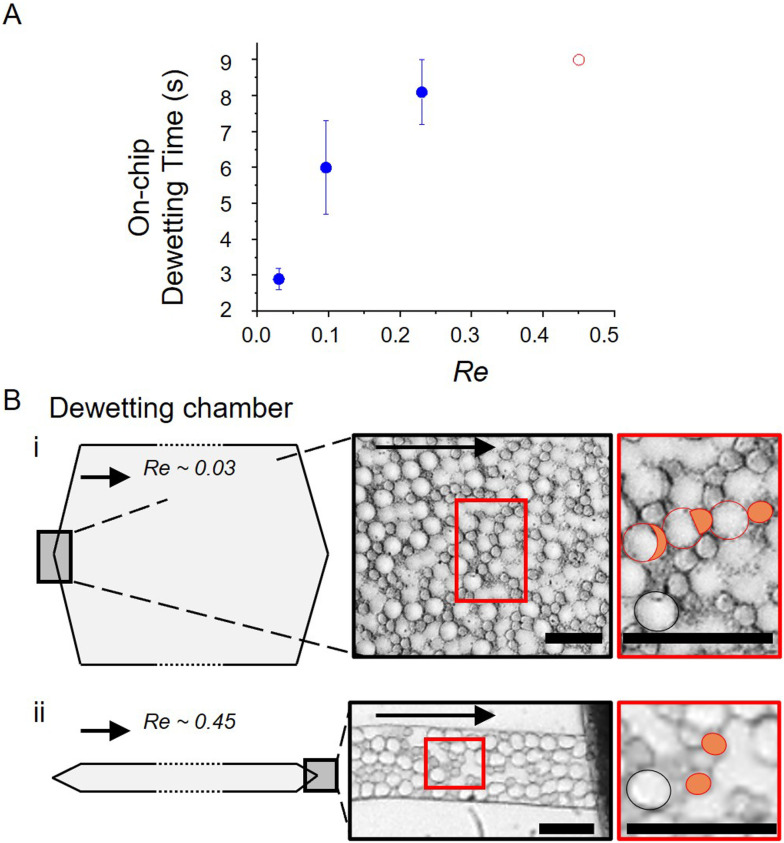
The influence of flow speed on on-chip dewetting dynamics for 2 : 1 DOPG : DOPC 25 mg mL^−1^, 1.2 confinement, low P188, and no salt. A) On-chip dewetting time as a function of Reynolds number (Re). Blue markers denote complete dewetting within the observed channel length. The red circle at Re ∼ 0.45 indicates the maximum residence time within the corresponding dewetting chamber (9 s), which is less than the required dewetting time. B) Morphology of double emulsions within the chamber at two different flow speeds. Arrows indicate flow direction. Red circles in the magnified inset indicate droplets undergoing phase separation, orange-filled circles highlight phase-separated octanol droplets, and black circles represent non-dewetted DEs. Scale bars are 30 μm. i) 1.2 mm s^−1^ and lower shear. ii) 19.2 mm s^−1^ and higher shear.

Having established the chemical viability of the 0.1% P188 regime, we next examined the influence of fluid dynamics to optimize production throughput. Contrary to the assumption that shear facilitates oil stripping,^14,33^ we found that dewetting is significantly more effective at lower flow speeds and lower shear rates. Fig. 6A illustrates the inverse relationship between the Reynolds number (Re) and dewetting efficiency. As Re increases, the on-chip dewetting time increases. At the highest speed tested (∼19 mm s^−1^; Re ≈ 0.45), the dewetting time exceeded 9 seconds, with a significant fraction of double emulsions failing to dewet entirely on chip. Technical constraints regarding flow resistance prevented testing of longer residence times at this speed, but the trend clearly demonstrates that high flow speeds under confinement impede the dewetting process.

Insight into this rate-limiting mechanism is provided by droplet morphology (Fig. 6B). At low speeds, droplets exhibit a characteristic accumulation of the octanol “pocket” at the leading edge, a precursor to separation. However, at high speeds, droplets undergo significant deformation and lack this anterior octanol pocket. We attribute this to a shear-induced Marangoni effect. Although the bulk surfactant concentration is low (0.1%), strong internal circulation drives surfactant accumulation toward the front of the droplet. This creates a local interfacial tension gradient that generates a backward-facing Marangoni stress, opposing the formation of the octanol pocket. This result suggests a nuance to the role of shear stress: while the confinement-induced shear is necessary to initiate dewetting,^52^ excessive shear with high flow speed amplifies dynamic stresses that counteract the thermodynamic driving force.

These fluid dynamic constraints define a clear design rule for maximizing throughput. In the original 5% P188 study,^9,11^ on-chip dewetting required minutes. This means accommodating longer residence times on-chip will require longer channels increasing flow resistance and limiting how high the pressure and therefore throughput can be. In contrast, the rapid kinetics of the 0.1% P188 regime (seconds) allow for a different scaling strategy. Because dewetting is favored by low shear but is inherently fast, throughput can be maximized by increasing the channel width rather than flow velocity. This “slow-flow, wide-channel” approach reduces hydraulic resistance while maintaining the low-shear environment necessary for reliable, rapid GUV formation.

### 4.5 Validation of biological functionality of the low surfactant/no glycerol regime

**Fig. 7:**
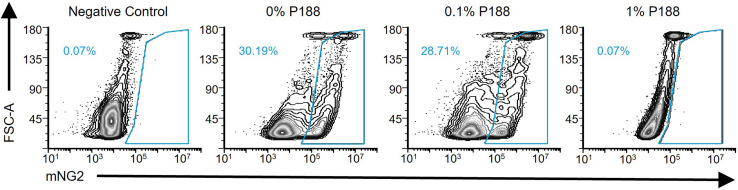
Effect of P188 on SARS-CoV-2 spike protein-mediated cell–cell fusion measured by a flow cytometry-based split mNeonGreen assay (see materials and methods). Percentages indicate the fraction of cells that have fused.

Having established the hydrodynamic rules for low-surfactant GUV production, we sought to confirm the biological utility of this regime by defining the maximum acceptable P188 concentration for use with sensitive biological systems. Previous studies report that high P188 concentrations can inhibit cell adhesion,^30^ reduce cell-free expression,^31^ and inhibit protein polymerization.^9^ The cell–cell fusion assay performed with SARS-CoV-2 spike protein and ACE2 -receptor-bearing cells revealed that concentrations typical of standard GUV protocols (5% P188) are indeed highly inhibitory (Fig. 7). Even 1% P188 completely inhibited fusion (0.07% success rate). This inhibition is consistent with P188′s ability to bind exposed hydrophobic domains of viral surface proteins^59^ and intercalate into membranes as a sealant,^20–23^ physically blocking the fusion machinery. These phenomena are expected to extend to other membrane proteins whose function depends on specific molecular interactions; therefore, we suspect that high concentrations of P188 affect the function of many membrane proteins. Conversely, our optimized 0.1% P188 condition maintained fusion rates (∼29%) comparable to the surfactant-free control (30%). This finding confirms that the high surfactant concentrations typically required for dewetting actively suppress complex membrane interactions. Relying on post-production washing is an insufficient strategy for surfactant removal. Similar to the persistence of Triton X-100 in membrane protein reconstitution,^60^ P188 adsorbs or inserts into lipid membranes and can resist complete removal even after a few culture passages.^30,61^

**Fig. 8:**
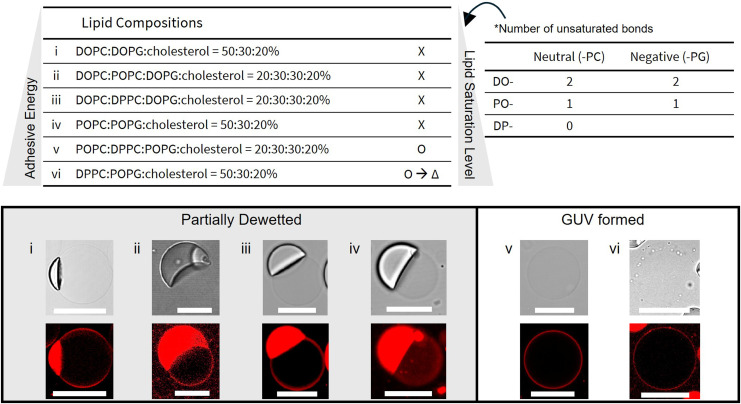
GUV generation under biologically relevant conditions. All conditions were prepared with 10 mM HEPES, 100 mM KCl, 290 mM glucose, 0.1% P188 and no glycerol in the OA with a lipid concentration of 25 mg mL^−1^. The specific lipid compositions are detailed in the left table, while the number of unsaturated bonds in neutral and negatively charged phospholipids is indicated in the right table. The state of GUVs (2000 × *g*, 1 min) post-centrifuged is classified as follows. “X”: partially dewetted, “O”: completely dewetted, or “O→Δ”: ompletely dewetted but later fragmented. For each condition, confocal images of both brightfield (upper panels) and fluorescence images (lower panels) of the post-centrifuged GUVs are shown. Scale bars: 10 μm.

Similar concerns apply to glycerol, which is known to increase lipid viscosity,^19^ alter protein functions,^15,27^ and inhibit *in vitro* transcription/translation (IVTT)^28,29^ at high concentrations. Membrane protein dynamics and function are highly sensitive to the membrane environment; therefore, we expect that proteins undergoing dynamic conformational changes could be affected by glycerol. Notably, while short-term cell survival is maintained at 1% P188 without glycerol, GUVs are destroyed under this condition, likely because they lack the structural proteins and cytoskeletal scaffolds necessary to withstand such harsh chemical environments. Therefore, generating GUVs at 0.1% P188 or lower is crucial for reliable GUV-based assays.

### 4.6 Influence of lipid adhesion energy on dewetting in physiological conditions

As shown in the previous sections, while inclusion of physiological salt and buffer concentrations is essential for maintaining protein structure and membrane stability, these components significantly hinder the thermodynamic process of on-chip dewetting at biologically tolerable surfactant levels. To reconcile the requirement for physiological compatibility with the physics of dewetting, we investigated whether modulating the lipid bilayer's adhesion energy could overcome the inhibitory effects of salt. Rather than focusing solely on process engineering, we systematically analyzed how lipid acyl chain saturation influences the energy balance required for successful GUV formation.

We hypothesized that increasing the packing density of the lipid bilayer would enhance the adhesion energy necessary to drive oil expulsion. A saturated lipid like DPPC (16 : 0) imparts significantly higher rigidity and tighter packing compared to POPC (16 : 0, 18 : 1) and DOPC (18 : 1), which contain one and two monounsaturated acyl chains, respectively (Fig. S11). To test this, we evaluated lipid compositions mimicking mammalian membranes, typically containing 20% cholesterol and 30% negatively charged lipids,^62,63^ across a spectrum of acyl chain saturation. We observed a clear threshold where membrane order dictated the success of dewetting. Compositions dominated by disordered, unsaturated lipids, specifically those rich in DOPC and POPC, failed to achieve complete dewetting, even when the highly disordered DOPC was entirely replaced by POPC. Successful oil exclusion was only achieved when the fraction of fully saturated DPPC was increased significantly, substituting 60% or 100% of the POPC content. This result shows that higher acyl chain saturation promotes the dewetting transition in high-salt environments.

Mechanistically, this dependence on saturation is best understood through the framework of free energy minimization and the Young–Dupré equation^64,65^ (Fig. S12):

1 *γ*_B_ = *γ*_m1_ + *γ*_m2_ − *ε*

where *γ*_B_ is the bilayer surface tension, *γ*_m1_ and *γ*_m2_ are the surface tensions between the middle oil phase and each of the inner and outer aqueous phases, and *ε* is the adhesion energy between the monolayer leaflets. For DE dewetting, *γ*_B_ corresponds to *γ*_13_, and eqn (1) adapts to *S*_2_ = −*ε*. Thus, bilayer formation is more favorable with higher adhesion energy. Saturated lipids like DPPC pack tightly, maximizing van der Waals interactions and increasing this adhesion energy. In contrast, the disordered packing of unsaturated DOPC retains more octanol, further hindering tighter lipid packing and lowering the adhesion energy.^66^ This picture aligns with results utilizing a droplet interface bilayer (DIB),^67^ where DOPC often fails to form stable bilayers but tighter-packing lipids, like DPhPC, readily succeed. Moreover, DIB research shows that the adhesion energy is maximized at 0–30% cholesterol,^64^ supporting our use of 20% cholesterol in the experiments.

However, maximizing saturation to force dewetting introduces a critical trade-off regarding long-term structural integrity. While the fully saturated composition with 100% DPPC substitution dewetted successfully, the resulting GUVs exhibited non-spherical morphologies and fragmented over several days. This instability likely arises from phase separation,^68,69^ driven by thermodynamic incompatibility between rigid DPPC (*T*_m_ = 41 °C) and the highly fluidic matrix (DOPC *T*_m_ = −17 °C; POPC *T*_m_ = −2 °C). This packing mismatch forces DPPC to segregate into an ordered microdomain to minimize free energy. A study on an analogous ternary system (DPPC/DPPG/cholesterol) confirm that such microdomains, particularly in the presence of charged lipids and cholesterol, can induce pore formation and membrane morphology changes.^70^ Indeed, GUVs with 100% DPPC substitution have non-spherical morphologies (Fig. S13A), indicating asymmetric surface stresses.^70^ Other potential mechanisms are GUV deflation *via* pore formation, followed by fission (Fig. S13B) due to the excess surface area at phase boundaries,^71^ though this requires further study.

The key takeaway is that the degree of saturation must be carefully balanced: condition 5, which contained lower DPPC and higher POPC molar fractions, successfully dewetted while yielding stable GUVs by minimizing large-scale domain formation.^68^ Overall, these results provide an essential guideline: successful production of stable GUVs under biorelevant conditions requires optimizing lipid composition to maximize adhesion energy while avoiding microdomain-induced instability.

## Conclusion

The controlled assembly of synthetic cells requires robust physical understanding of GUV formation. To address this need, we conducted the first systematic investigation of dewetting dynamics in a widely used DE-based production method, bridging the gap between current empirical approaches and predictive design. Our results establish that successful dewetting is determined by a critical balance between dynamic flow-field stresses and thermodynamic forces. First, we establish that confinement is essential for rapid dewetting. We then identify distinct regimes governed by surfactant concentration. High P188 conditions generate opposing Marangoni stresses and necessitate the addition of glycerol to prevent rupture. Low P188 concentrations minimize this dynamic barrier, and other thermodynamic factors become the dominant control parameters. Specifically, in the absence of salt, lipid concentration and charge act synergistically to promote dewetting. In contrast, under biologically relevant conditions containing salt, lipid adhesion energy emerges as the critical parameter; here, increasing membrane packing *via* saturation is required to counterbalance the inhibitory effects of salt. Moreover, we demonstrate how understanding shear-induced surfactant redistribution under confined flow can be used to maximize throughput. This systematic study transforms a complex set of variables into practical guidelines, making the popular DE method significantly more robust and adaptable. A key secondary outcome is the ability to generate GUVs in biocompatible conditions by eliminating reliance on problematic additives like high glycerol and P188 and including physiologically relevant buffer concentrations. By reconciling microfluidic dynamics with membrane biophysics, this work establishes a reliable and scalable strategy for advancing GUV platforms in biophysical and biomedical applications.

## Conflicts of interest

There are no conflicts to declare.

## Supplementary Material

LC-026-D6LC00098C-s001

## Data Availability

The data supporting this article have been included as part of the supplementary information (SI). Supplementary information: including designs of the microfluidic drop-making device, additional micrographs of dewetting states and GUV formation, experimental data for on-chip dewetting times, and detailed interfacial tension measurements across varied lipid compositions and surfactant concentrations. It also provides thermodynamic analysis of dewetting, Marangoni stress estimations, and gating strategies for flow cytometry-based quantification. See DOI: https://doi.org/10.1039/d6lc00098c.

## References

[cit1] Sharma B., Moghimianavval H., Hwang S.-W., Liu A. P. (2021). Membranes.

[cit2] Dimova R. (2019). Annu. Rev. Biophys..

[cit3] Litschel T., Schwille P. (2021). Annu. Rev. Biophys..

[cit4] Bailoni E., Partipilo M., Coenradij J., Grundel D. A. J., Slotboom D. J., Poolman B. (2023). ACS Synth. Biol..

[cit5] Imai M., Sakuma Y., Kurisu M., Walde P. (2022). Soft Matter.

[cit6] Ugrinic M., deMello A., Tang T.-Y. D. (2019). Chem.

[cit7] Ai Y., Xie R., Xiong J., Liang Q. (2020). Small.

[cit8] Weiss M., Frohnmayer J. P., Benk L. T., Haller B., Janiesch J.-W., Heitkamp T., Börsch M., Lira R. B., Dimova R., Lipowsky R., Bodenschatz E., Baret J.-C., Vidakovic-Koch T., Sundmacher K., Platzman I., Spatz J. P. (2018). Nat. Mater..

[cit9] Deshpande S., Caspi Y., Meijering A. E. C., Dekker C. (2016). Nat. Commun..

[cit10] Al Nahas K., Fletcher M., Hammond K., Nehls C., Cama J., Ryadnov M. G., Keyser U. F. (2022). Anal. Chem..

[cit11] Tivony R., Fletcher M., Al Nahas K., Keyser U. F. (2021). ACS Synth. Biol..

[cit12] Al Nahas K., Cama J., Schaich M., Hammond K., Deshpande S., Dekker C., Ryadnov M. G., Keyser U. F. (2019). Lab Chip.

[cit13] Fanalista F., Birnie A., Maan R., Burla F., Charles K., Pawlik G., Deshpande S., Koenderink G. H., Dogterom M., Dekker C. (2019). ACS Nano.

[cit14] Bao P., Paterson D. A., Peyman S. A., Jones J. C., Sandoe J. A. T., Gleeson H. F., Evans S. D., Bushby R. J. (2021). Soft Matter.

[cit15] Guerzoni L. P. B., De Goes A. V. C., Kalacheva M., Haduła J., Mork M., De Laporte L., Boersma A. J. (2022). Adv. Sci..

[cit16] Ushiyama R., Koiwai K., Suzuki H. (2022). Sens. Actuators, B.

[cit17] Schaich M., Sobota D., Sleath H., Cama J., Keyser U. F. (2020). Biochim. Biophys. Acta, Biomembr..

[cit18] Teh S.-Y., Khnouf R., Fan H., Lee A. P. (2011). Biomicrofluidics.

[cit19] Pocivavsek L., Gavrilov K., Cao K. D., Chi E. Y., Li D., Lin B., Meron M., Majewski J., Lee K. Y. C. (2011). Biophys. J..

[cit20] Wang J.-Y., Chin J., Marks J. D., Lee K. Y. C. (2010). Langmuir.

[cit21] Wang J., Segatori L., Biswal S. L. (2014). Soft Matter.

[cit22] Van Zee N. J., Peroutka A. S., Hillmyer M. A., Lodge T. P. (2023). Langmuir.

[cit23] Wu G., Majewski J., Ege C., Kjaer K., Weygand M. J., Lee K. Y. C. (2005). Biophys. J..

[cit24] Lundbæk J. A., Birn P., Girshman J., Hansen A. J., Andersen O. S. (1996). Biochemistry.

[cit25] Bernard C., Carotenuto A. R., Pugno N. M., Deseri L., Fraldi M. (2024). Meccanica.

[cit26] Gohrbandt M., Lipski A., Grimshaw J. W., Buttress J. A., Baig Z., Herkenhoff B., Walter S., Kurre R., Deckers-Hebestreit G., Strahl H. (2022). EMBO J..

[cit27] Wang M., Sheng Y., Cui H., Li A., Li X., Huang H. (2022). ACS Sustainable Chem. Eng..

[cit28] Promega Corporation , 2015

[cit29] Warfel K. F., Williams A., Wong D. A., Sobol S. E., Desai P., Li J., Chang Y.-F., DeLisa M. P., Karim A. S., Jewett M. C. (2023). ACS Synth. Biol..

[cit30] Tharmalingam T., Ghebeh H., Wuerz T., Butler M. (2008). Mol. Biotechnol..

[cit31] Ho K. K. Y., Lee J. W., Durand G., Majumder S., Liu A. P. (2017). PLoS One.

[cit32] Ushiyama R., Nanjo S., Tsugane M., Sato R., Matsuura T., Suzuki H. (2023). ACS Synth. Biol..

[cit33] Schaich M., Cama J., Al Nahas K., Sobota D., Sleath H., Jahnke K., Deshpande S., Dekker C., Keyser U. F. (2019). Mol. Pharmaceutics.

[cit34] Narayanappa A. T., Mwilu S., Holdread S., Hammett K., Bu G., Dodson E. C., Brooks J. W. (2019). BioTechniques.

[cit35] Abate A. R., Thiele J., Weinhart M., Weitz D. A. (2010). Lab Chip.

[cit36] Deshpande S., Birnie A., Dekker C. (2017). Biomicrofluidics.

[cit37] Hinna A., Steiniger F., Hupfeld S., Stein P., Kuntsche J., Brandl M. (2016). J. Liposome Res..

[cit38] Matreyek K. A., Stephany J. J., Chiasson M. A., Hasle N., Fowler D. M. (2019). Nucleic Acids Res..

[cit39] Tan T. J. C., Mou Z., Lei R., Ouyang W. O., Yuan M., Song G., Andrabi R., Wilson I. A., Kieffer C., Dai X., Matreyek K. A., Wu N. C. (2023). Nat. Commun..

[cit40] Deng N.-N., Yelleswarapu M., Huck W. T. S. (2016). J. Am. Chem. Soc..

[cit41] Torza S., Mason S. G. (1969). Science.

[cit42] Deng N.-N., Wang W., Ju X.-J., Xie R., Weitz D. A., Chu L.-Y. (2013). Lab Chip.

[cit43] Kong T., Liu Z., Song Y., Wang L., Shum H. C. (2013). Soft Matter.

[cit44] Schmitt M., Stark H. (2016). Phys. Fluids.

[cit45] Hester E. W., Carney S., Shah V., Arnheim A., Patel B., Di Carlo D., Bertozzi A. L. (2023). Proc. Natl. Acad. Sci. U. S. A..

[cit46] Choi S. B., Park J. Y., Moon J. Y., Lee J. S. (2018). Phys. Rev. E.

[cit47] Gannon A., Quaife B., Young Y.-N. (2024). Soft Matter.

[cit48] Soligo G., Roccon A., Soldati A. (2020). Meccanica.

[cit49] Stan C. A., Ellerbee A. K., Guglielmini L., Stone H. A., Whitesides G. M. (2013). Lab Chip.

[cit50] Jing W., Han H.-S. (2022). Anal. Chem..

[cit51] Nanjo S., Tsugane M., Matsuura T., Suzuki H. (2025). Electrical Engineering in Japan.

[cit52] Pannacci N., Bruus H., Bartolo D., Etchart I., Lockhart T., Hennequin Y., Willaime H., Tabeling P. (2008). Phys. Rev. Lett..

[cit53] Hayward R. C., Utada A. S., Dan N., Weitz D. A. (2006). Langmuir.

[cit54] Ji J. and Kawano R., ChemRxiv, 2024, preprint, 10.26434/chemrxiv-2024-4gnff-v2

[cit55] Böckmann R. A., Hac A., Heimburg T., Grubmüller H. (2003). Biophys. J..

[cit56] De Mel J. U., Gupta S., Perera R. M., Ngo L., Zolnierczuk P., Bleuel M., Pingali S. V., Schneider G. J. (2020). Langmuir.

[cit57] Yandrapalli N., Petit J., Bäumchen O., Robinson T. (2021). Commun. Chem..

[cit58] Kang Z., Zhu P., Kong T., Wang L. (2016). Micromachines.

[cit59] Lee R. C. , Ling M. X., Nguyen M., Mccollum K. J. and Bigdelle V. A., WO2022051713A1, 2022

[cit60] Rigaud J.-L., Pitard B., Levy D. (1995). Biochim. Biophys. Acta, Bioenerg..

[cit61] Palomares L. A., González M., Ramírez O. T. (2000). Enzyme Microb. Technol..

[cit62] Lorent J. H., Levental K. R., Ganesan L., Rivera-Longsworth G., Sezgin E., Doktorova M., Lyman E., Levental I. (2020). Nat. Chem. Biol..

[cit63] Naito T., Ercan B., Krshnan L., Triebl A., Koh D. H. Z., Wei F.-Y., Tomizawa K., Torta F. T., Wenk M. R., Saheki Y. (2019). eLife.

[cit64] El-Beyrouthy J., Makhoul-Mansour M. M., Taylor G., Sarles S. A., Freeman E. C. (2019). J. R. Soc. Interface.

[cit65] Schrader M. E. (1995). Langmuir.

[cit66] Elliott J. R., Haydon D. A. (1979). Biochim. Biophys. Acta, Biomembr..

[cit67] Venkatesan G. A., Taylor G. J., Basham C. M., Brady N. G., Collier C. P., Sarles S. A. (2018). Biomicrofluidics.

[cit68] Garvey C. J., Bryant S. J., Elbourne A., Hunt T., Kent B., Kreuzer M., Strobl M., Steitz R., Bryant G. (2023). J. Colloid Interface Sci..

[cit69] Vu T. Q., Peruzzi J. A., Sant'Anna L. E., Roth E. W., Kamat N. P. (2022). Nano Lett..

[cit70] Himeno H., Ito H., Higuchi Y., Hamada T., Shimokawa N., Takagi M. (2015). Phys. Rev. E:Stat., Nonlinear, Soft Matter Phys..

[cit71] Dreher Y., Jahnke K., Bobkova E., Spatz J. P., Göpfrich K. (2021). Angew. Chem..

