# Supplementary material for: Systematic investigation of double emulsion dewetting dynamics for the robust production of giant unilamellar vesicles
Source: Lab Chip. 2026 Apr 22;26(10):3039–53. doi: 10.1039/d6lc00098c (PMC13102286; doi:10.1039/d6lc00098c)
Supplement: LC-026-D6LC00098C-s001 [file LC-026-D6LC00098C-s001.pdf]

Supplementary Information (SI) for Lab on a Chip.
This journal is © The Royal Society of Chemistry 2026

Electronic Supplementary Material (ESI) for Lab on a Chip.

Part I: Supplementary Figures S1-S14

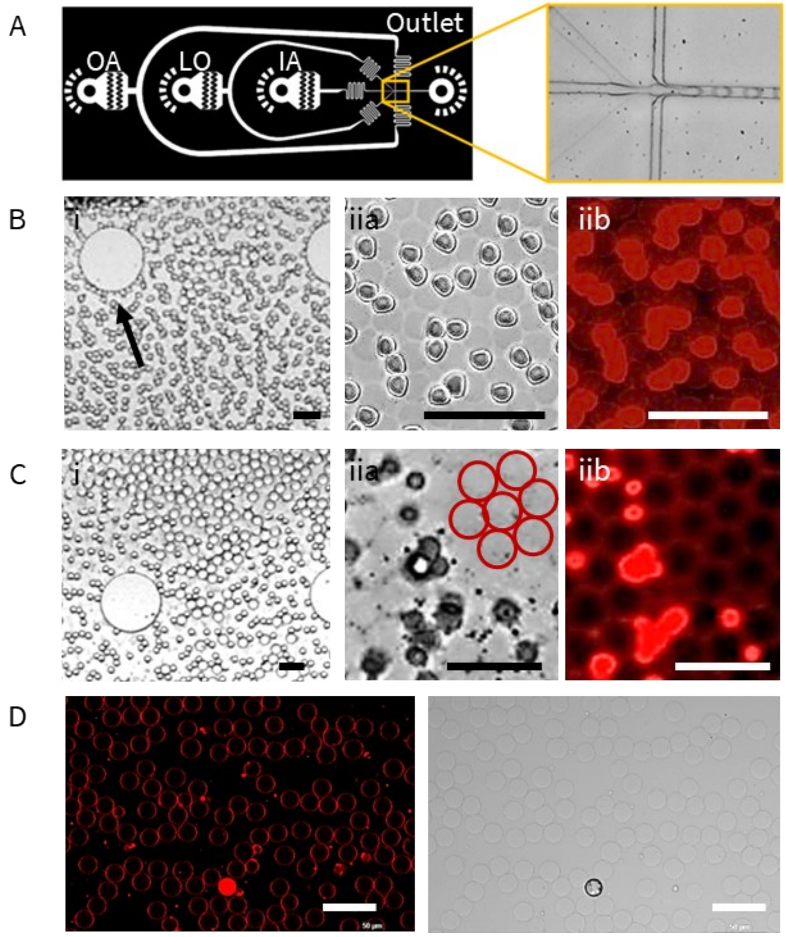

Supplementary Fig. S1 (A) Schematic of drop maker with inlets for inner aqueous (IA), lipid oil (LO), outer aqueous (OA) phases. (B) Representative high-speed camera micrograph of the dewetting channel under conditions of partial dewetting. i) The left panel shows double emulsions with intact shells (circles) and those in a partially dewetted state (dark crescents); the black arrow indicates a structural support post. iia-b) The right panel provides a higher-magnification wide-field views of these states using bright-field (a) and fluorescence (b) channels. (C) Corresponding brightfield image in the dewetting channel under conditions of complete on-chip dewetting. i) The left panel displays the distribution of double emulsions and GUVs relative to detached octanol droplets. iia-b) The right panel (higher magnification) reveals GUVs situated between larger octanol droplets (dark outlines). These GUVs are identified by the arrangement of smaller satellite octanol droplets along their perimeters; red circles highlight a honeycomb cluster of GUVs defined by these satellite droplets. (D) Representative confocal images of dewetted GUVs on-chip. Scale bar: 50 µm.

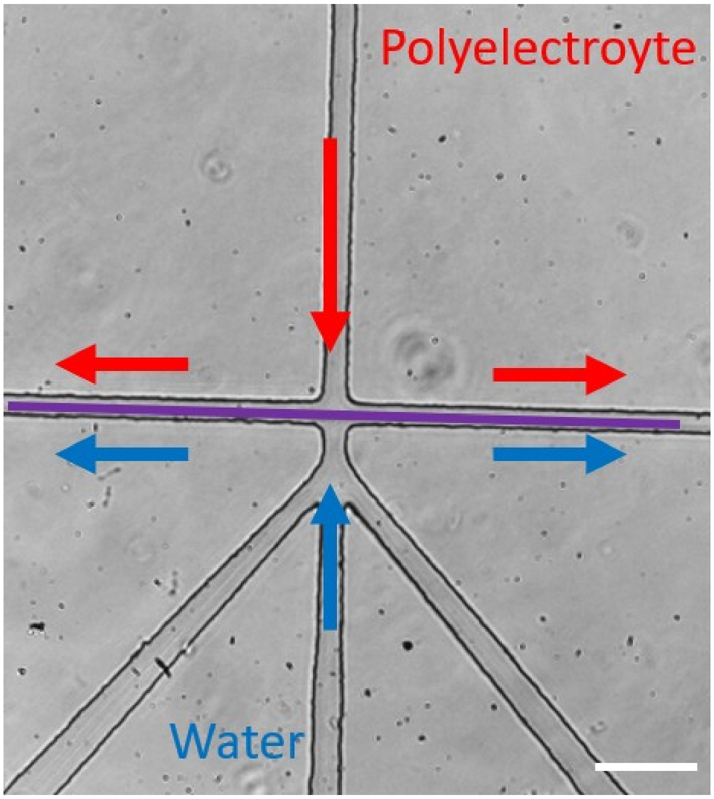

Supplementary Fig. S2 Micrograph illustrating flow confinement at a junction for selective surface treatment with polyelectrolyte solutions. The region above the purple line must be hydrophilic and the region below must be hydrophobic. Red arrows denote the polyelectrolyte solution flow path, and blue arrows denote the water flow path used for spatial restriction. Scale bar: 50 µm.

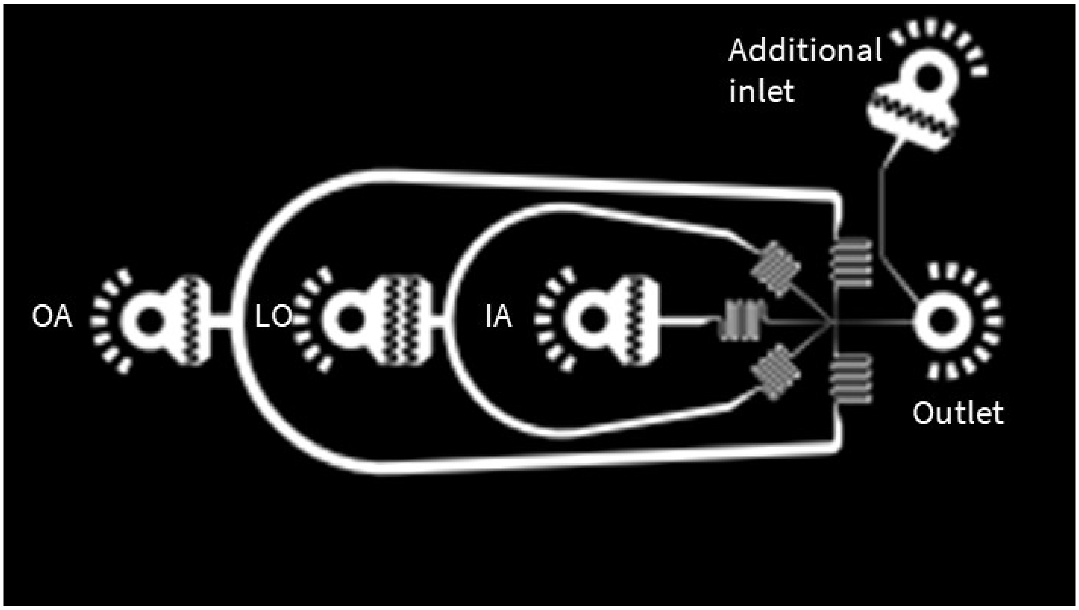

Supplementary Fig. S3 Diagram showing a side channel used to add high concentration P188 after drop-making to avoid altering fluid parameters at the junctions. The solutions mix as they rise through the tubing connecting the drop generator to the dewetting chamber.

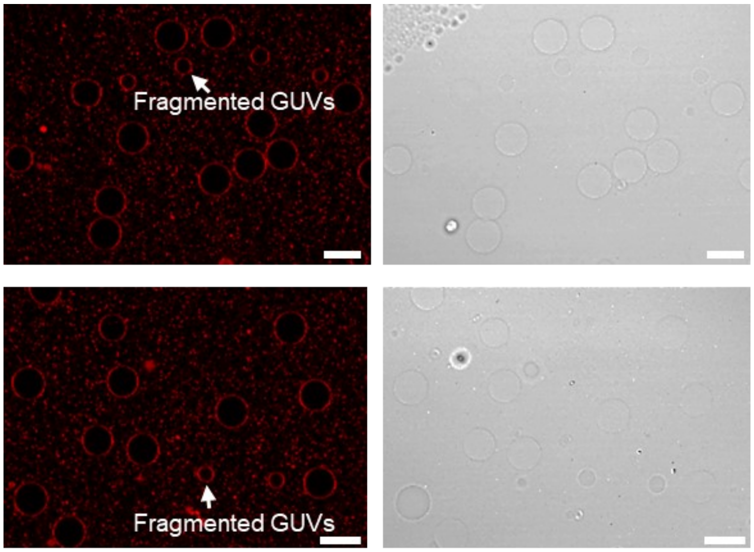

Supplementary Fig. S4 Confocal images of GUV fragmentation after centrifugation at 10000 × *g* for 1 min. Samples were prepared with 15 mg/mL of DOPC DOPG 2:1 mol% (LO) and 0.1% P188 (OA). Scale bar: 20 µm.

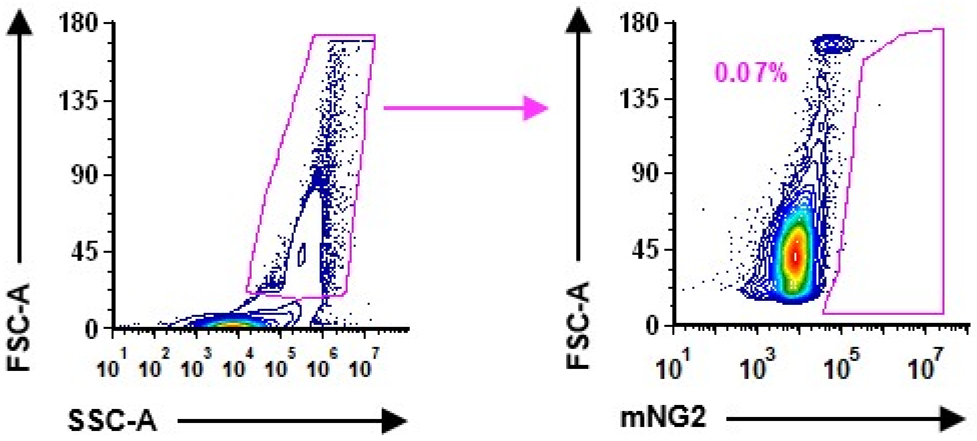

Supplementary Fig. S5 Gating strategy for flow cytometry-based quantification of successfully fused cells. Left plot displays gating for general cell population, and the right plot shows gating for fused cells (mNG2^{+}).

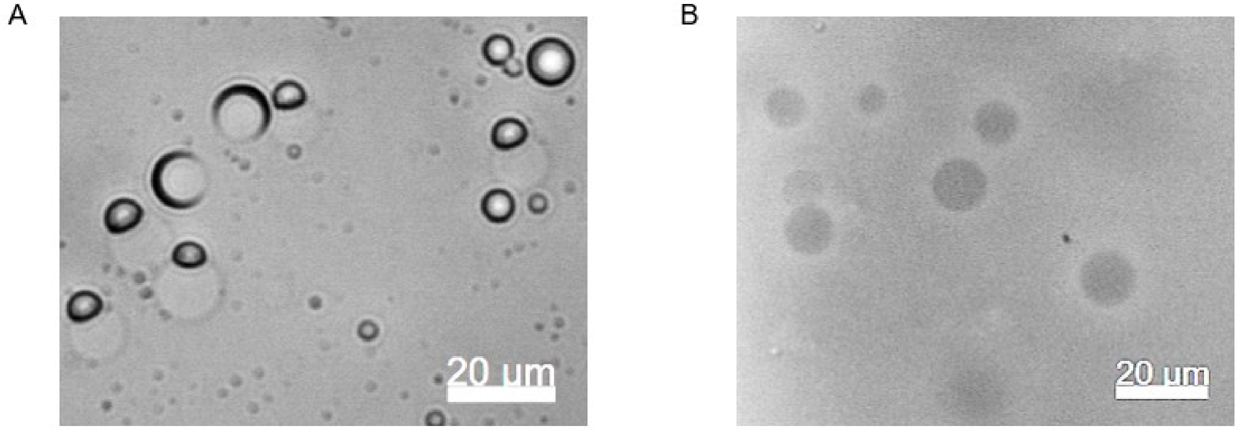

Supplementary Fig. S6 Brightfield images of GUVs on a hemocytometer prepared with 10% glycerol at high concentration of P188. (A) Partially dewetted GUVs and (B) GUVs visualized in the presence of glycerol. All conditions were prepared with 25 mg/mL 2:1 DOPC:DOPG (LO); 500 mM sucrose and 0.02% P188 at pH 7.6 (IA); 500 mM glucose, 10% glycerol, and 5% P188 at pH 7.6 (OA). Scale bar: 20 µm.

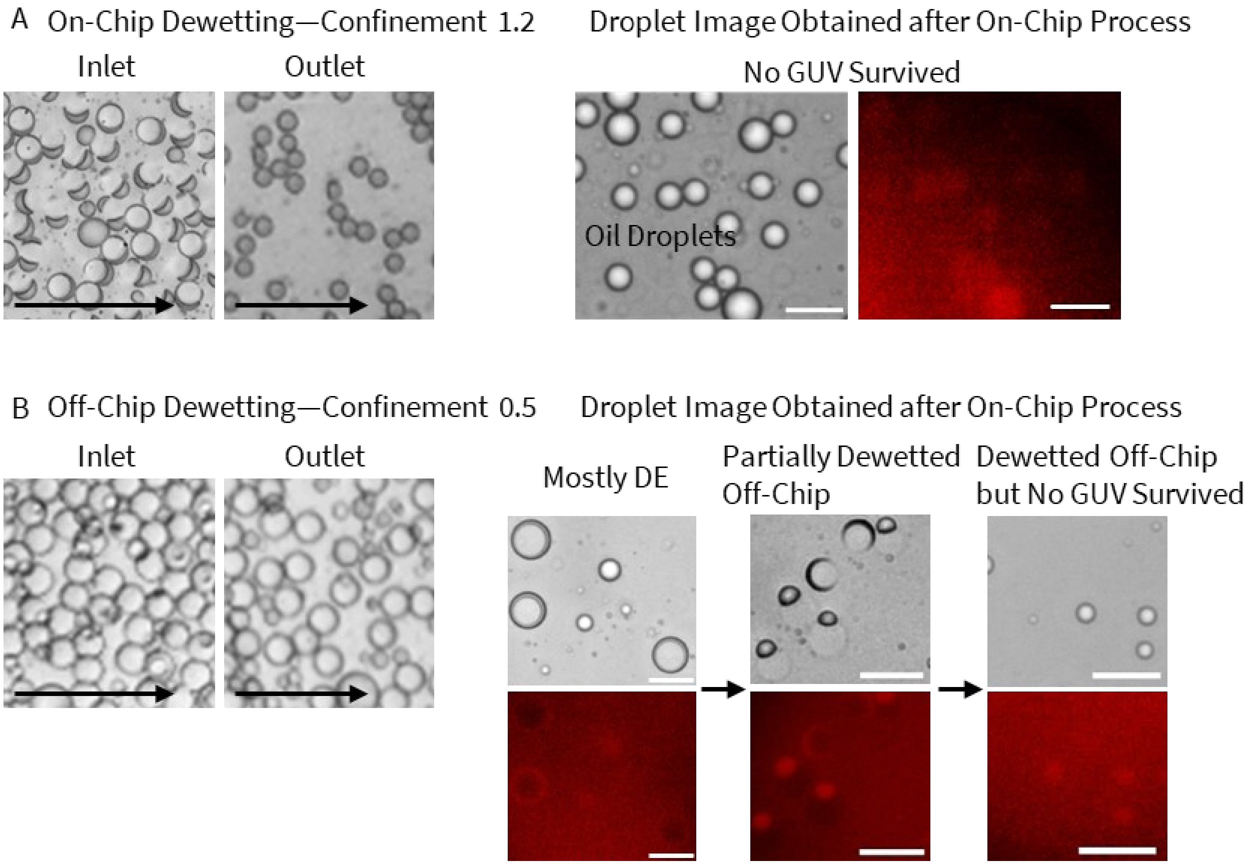

Supplementary Fig. S7 (A) On-chip or (B) off-chip dewetting outcomes at high concentration of P188 without glycerol. Left panels show droplets at the inlet and outlet of the dewetting chamber under specified confinement. Right panels include bright-field and fluorescent images of droplet outcomes post-dewetting. (A) On-chip dewetting resulted in no GUV survival, leaving only octanol droplets. (B) Off-chip DEs spontaneously dewetted but still ruptured. All conditions were prepared with 25 mg/mL 2:1 DOPC:DOPG (LO); 500 mM sucrose and 0.02% P188 at pH 7.6 (IA); 500 mM glucose and 5% P188 at pH 7.6 (OA). Scale bar: 20 µm.

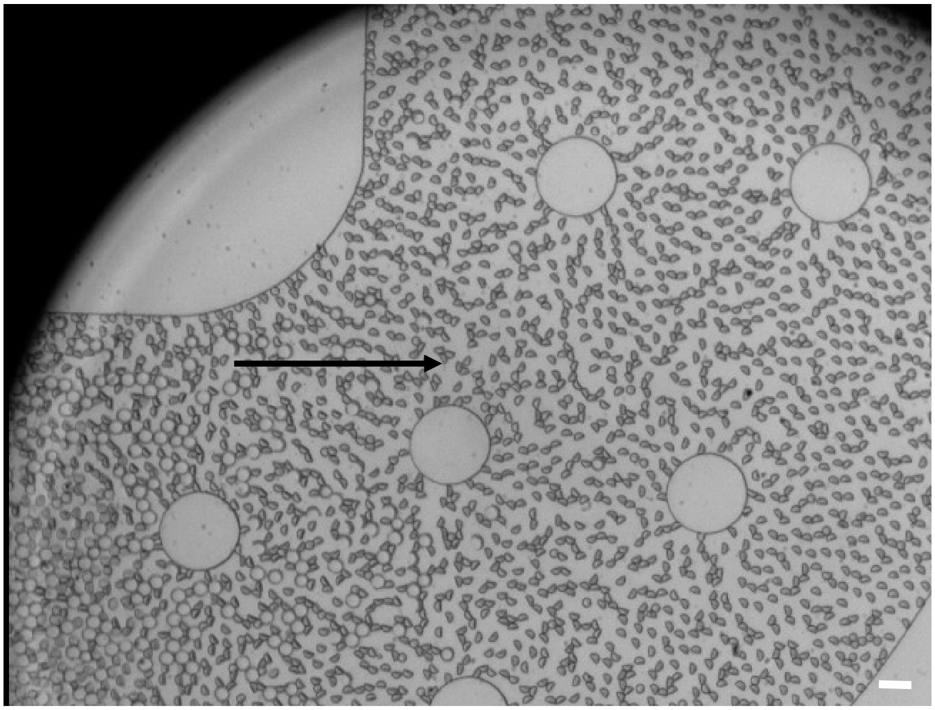

Supplementary Fig. S8 Brightfield image of double emulsions entering a dewetting channel with confinement >1. Confinement forces a partially dewetted state near the inlet. Scale bar: 50 µm.

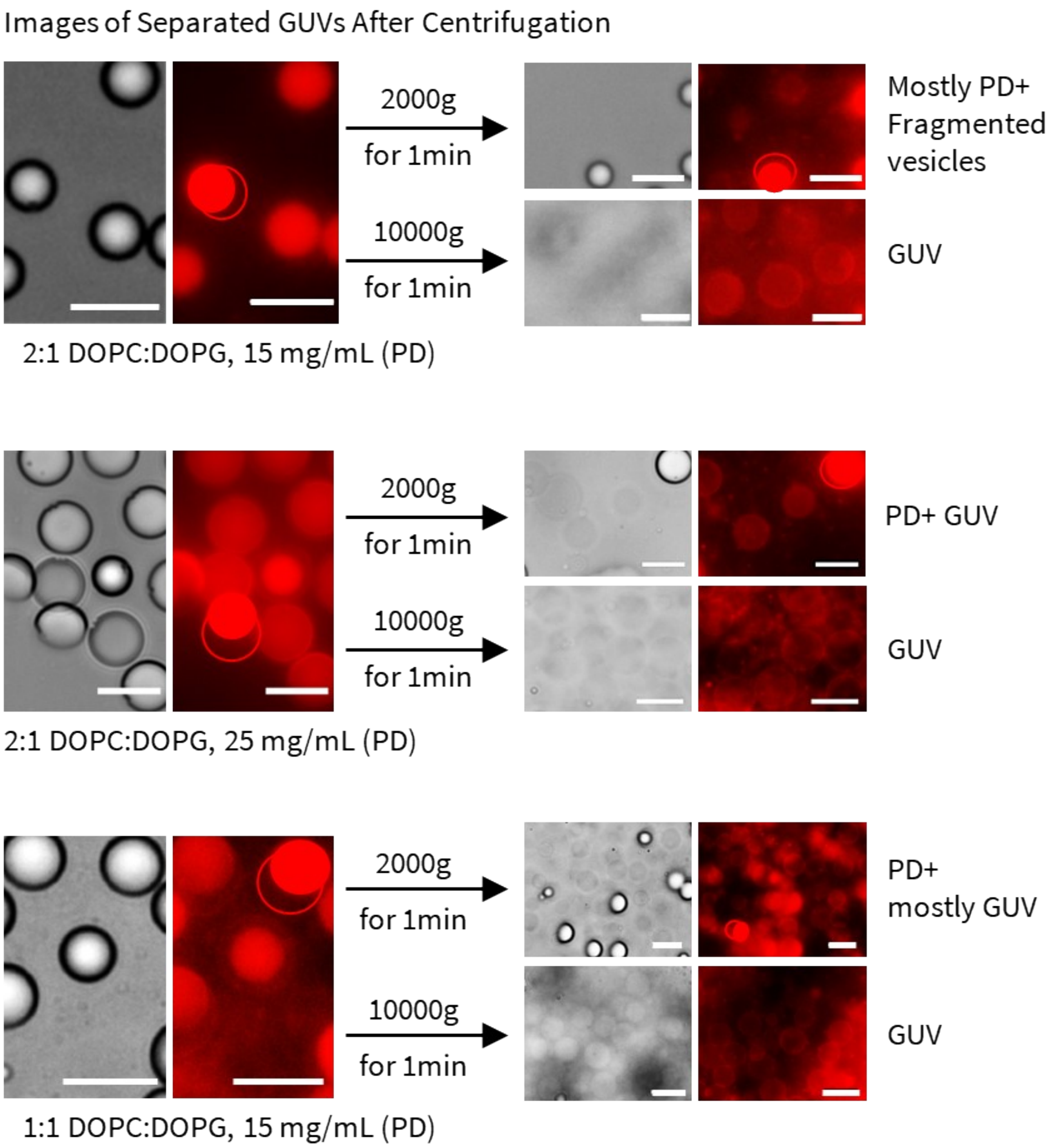

Supplementary Fig. S9 Fluorescence images of GUVs following centrifugation at 2000 × *g* and 10000 × *g* for 1 minute. All conditions were prepared with 500 mM glucose and 0.1% P188 in OA, along with varied DOPC and DOPG ratios and concentrations. Red circles highlight partially dewetted vesicles with attached oil droplets. Scale bar: 20 µm

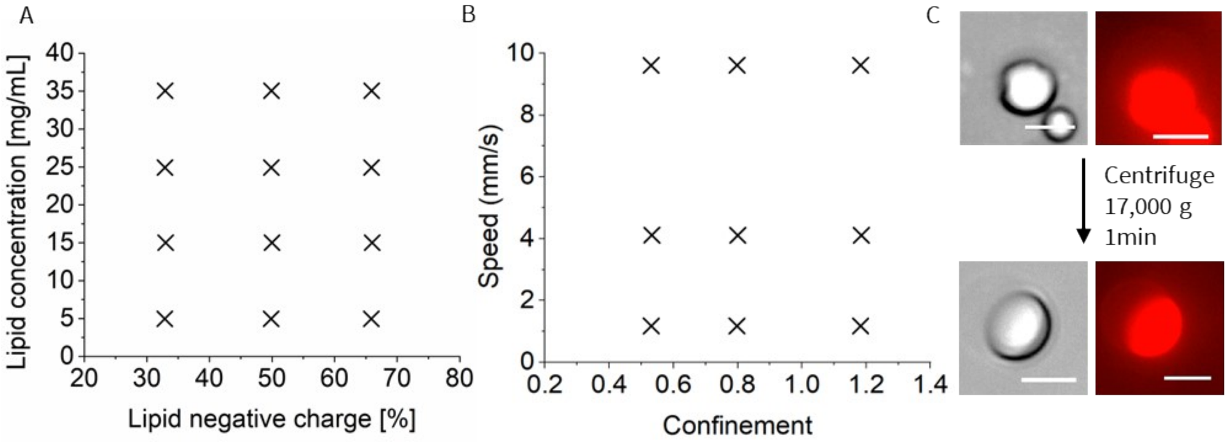

Supplementary Fig. S10 Dewetting inhibition by salt and buffer. (A) and (B) Experimental parameter space for lipid negative charge, concentration, flow speed, and confinement in the presence of 10 mM HEPES, 100 mM NaCl, and 0.1% P188. (C) Brightfield and fluorescence images of lipid structures post-centrifugation (17,000 × *g*, 1 min) for 35 mg/mL 2:1 DOPG:DOPC samples. Scale bar: 10 µm.

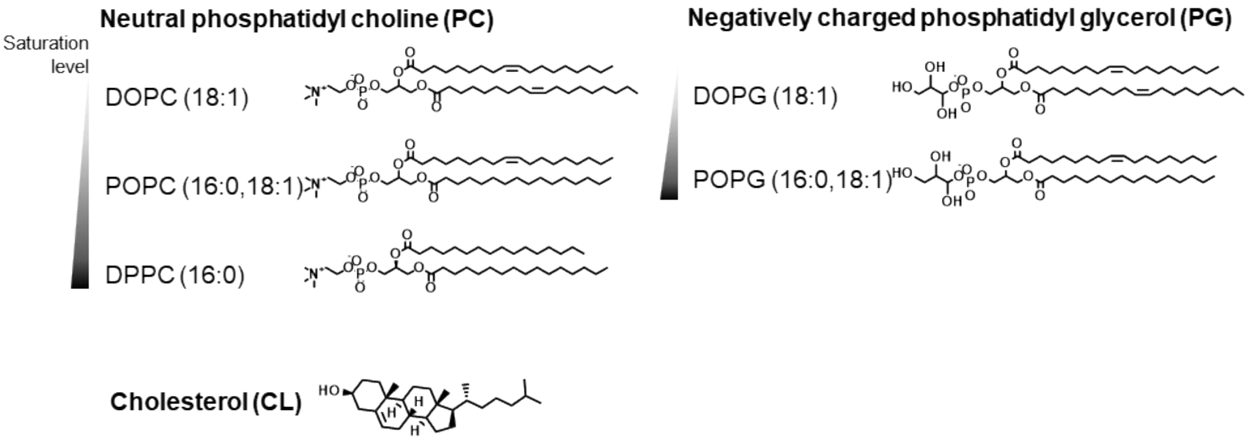

Supplementary Fig. S11 Chemical structures of neutral phosphatidylcholines (DOPC, POPC, DPPC), negatively charged phosphatidylglycerols (DOPG, POPG), and cholesterol (CL) used in this study.

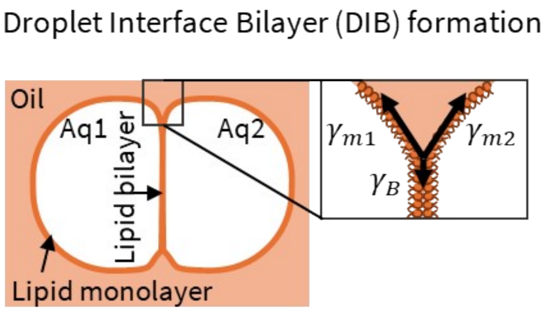

Supplementary Fig. S12 A schematic illustration of the formation of droplet interface bilayers (DIBs) between two aqueous droplets in lipid-stabilized oil. γ_{m1}, γ_{m2}, and γ_B represent the interfacial tensions acting at the monolayer and bilayer interfaces.

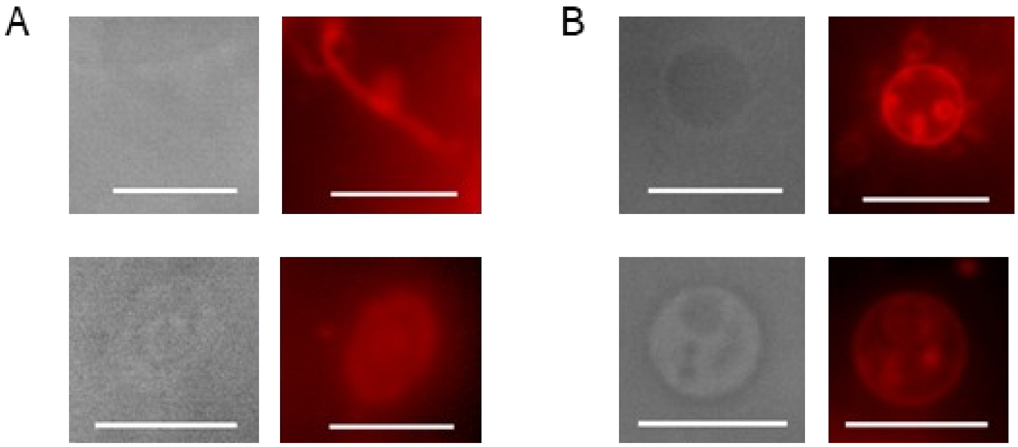

Supplementary Fig. S13 Brightfield and fluorescence images of GUVs prepared with 25 mg/mL 5:3:2 DPPC/POPG/cholesterol (LO) after centrifugation at 2000 × *g* for 1 minute. Panels show (A) non-spherical morphologies and (B) fragmented vesicles. Scale bar: 20 µm.

Part II. Supplementary Experimental Details & Figures S14-S16 & Tables

Thermodynamics of Dewetting

The dewetting outcomes at thermodynamic equilibrium are described by the spreading coefficient (S)[1–3], which is defined as:

$$S_i = \gamma_{jk} - (\gamma_{ij} + \gamma_{ik}) \tag{1}$$

where phases are defined as follows: IA (inner aqueous phase): 1, LO (lipid oil phase): 2, and OA (outer aqueous phase): 3. For dewetting to occur, the following three inequalities, which describe the relative surface energy densities (interfacial tension), must be satisfied.

1. The LO phase must resist spreading over the other phases:

$$S_2 = \gamma_{13} - \gamma_{12} - \gamma_{23} < 0 \tag{2}$$

2. The OA phase must favor spreading over the other phases, but not the IA phase:

$$S_1 = \gamma_{23} - \gamma_{12} - \gamma_{13} < 0 \tag{3}$$

$$S_3 = \gamma_{12} - \gamma_{13} - \gamma_{23} > 0 \tag{4}$$

Specifically, a configuration favors complete dewetting when the interfacial energy of the IA/LO interface (γ_{12}) is high enough that the system can lower its total energy by replacing that interface with the OA/LO (γ_{13}) and the IA/OA (γ_{23}) interfaces. Therefore, complete dewetting is thermodynamically favored when the surface tension between the IA and LO (γ_{12}) exceeds the sum of the surface tension between the OA and LO (γ_{23}) and that between the IA and OA phases (γ_{13}). This has been achieved using a higher concentration of the surfactant Pluronic F-68 (P188) in the OA phase to minimize γ_{13}, ensuring the formation of a separate LO phase rather than a stable LO shell.

Surface Tension and Adhesion Measurements

Surface tensions are dictated by the concentrations of P188 in the aqueous phases, the lipid compositions in the LO phase, and the presence of salts. We measured the surface tension between the LO and aqueous phases (γ_{12} and γ_{23}) using pendant droplet tensiometry (Tables S2 and S3). We used the model lipids DOPC (neutral) and DOPG (negative) at 2:1, 1:1, and 1:2 molar ratios while varying their concentrations from 5 to 35 mg/mL. As expected, increasing surfactant and lipid concentration decreases the surface tensions. Additionally, we observed that an increase in lipid negative charge further decreases these surface tensions, while adding salt generally increases them.

However, a complete calculation of the spreading coefficient was not possible for two reasons. We could not measure the surface tension between the two miscible IA and OA phases (γ_{13}).

Some aqueous-LO tensions were too low to form a proper pendant drop. Literature values for bilayer tension of GUVs embedding neutral lipid oil droplets range from 0.001 to 10 mN/m[4] while interfacial tensions between aqueous two-phase systems are typically in the range of 0.001-0.1 mN/m[5,6].

To circumvent these limitations, we employed droplet interface bilayer (DIB) measurements to assess the work of adhesion between oil-water interfaces[3]. We used green-labeled IA droplets (0.02% P188) and OA droplets (0.1% or 5% P188) dispersed in oil. These droplets were then brought into contact to form a bilayer. The resulting contact angle is governed by the Neumann triangle and Young-Dupré equation (Supplementary Figure S14):

$$W_a = \gamma_{12} + \gamma_{23} - \gamma_{13} \tag{5}$$

$$\textit{if } \theta \in \left(\frac{\pi}{2}, \pi\right) \qquad \gamma_{13} = -\gamma_{12}cos\theta_1 - \gamma_{23}cos\theta_2 \tag{6}$$

$$S_2 = - W_a \tag{7}$$

The contact angle approaching 90° indicates strong adhesion and favorable spreading coefficient S_2 ($S_2 < 0$). Conversely, when droplets failed to adhere ($\theta_1, \theta_2 = \pi$), we assumed $\Delta F \leq 0$.

DIB measurements confirmed the effects of lipid concentration, charge and salt. Interestingly, mimicking the high surfactant regime triggered interfacial instability (rupture, Supplementary Figure S15) upon contact between low surfactant (IA) and high surfactant (OA) droplets. This mirrors the instability observed during dewetting process. In the low surfactant regime (Supplementary Figure S16), increasing lipid concentration and charge enhanced the adhesion while adding salt impeded it, consistent with our dewetting results. Equation (6) implies that higher lipid concentration and charge, and the absence of salt correspondingly reduce γ_{13} as θ decreases in addition to reduction of γ_{12} and γ_{23} (Tables S2 and S3). Therefore, lipid concentration, charge, and ionic environment fundamentally alter the thermodynamic energy landscape of the dewetting process.

Marangoni Stress Estimation

The impact of Marangoni stress was estimated using the Marangoni number:

$$Ma = \Delta\gamma a/(\eta D) \tag{8}$$

The characteristic length scale is the droplet diameter (a) of 10 µm, and we use the dynamic viscosity (η) of water. Although we do not know the exact diffusion coefficients (D), we can estimate using the diffusion coefficient of approximately 10 µm²/s based on P188[7] and monolayer lipids[8], which is an upper end estimate as bilayer lipids having been measures with a

diffusion coefficient that is an order of magnitude lower[9]. Finally, assuming the mean surface tension range is 0.001 to 10 mN/m as discussed previously, even a conservative 1% difference estimate for $\Delta\gamma$ would yield a *Ma* in the range of 1−10^4, though it is more reasonable to estimate a mean surface tension of in the range of ~0.01 to 1 mN/m given our measurements (Tables S2-S3), which corresponds to a *Ma* of ~10-100, indicating that Marangoni effects are sufficiently strong to influence dewetting dynamics.

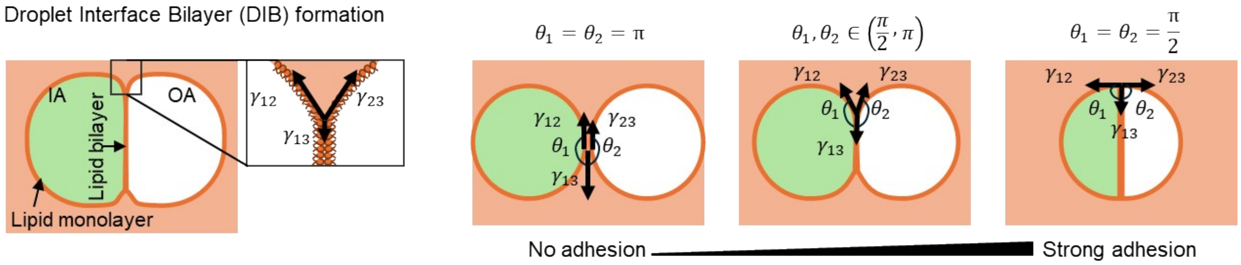

Supplementary Fig. S14 Analysis of interfacial energies via DIB formation. Illustrations show contact angles (θ) formed between IA and OA droplets dispersed in lipid-oil at varied levels of adhesion.

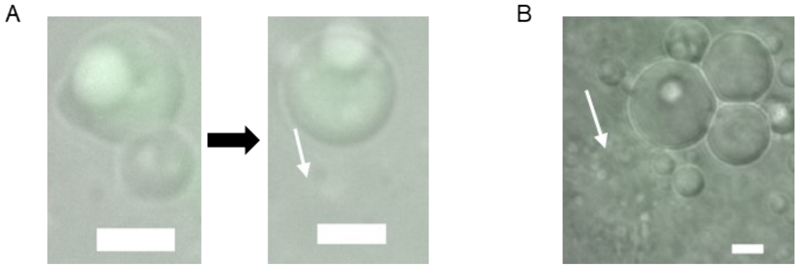

Supplementary Fig. S15 Representative brightfield images, with colocalized fluorescence, showing droplet rupture at contact in the high P188 regime. The white arrows indicate the rupture event captured in (A) time-lapse sequence before and after droplet rupture and (B) during droplet rupture. Scale bar: 10 µm.

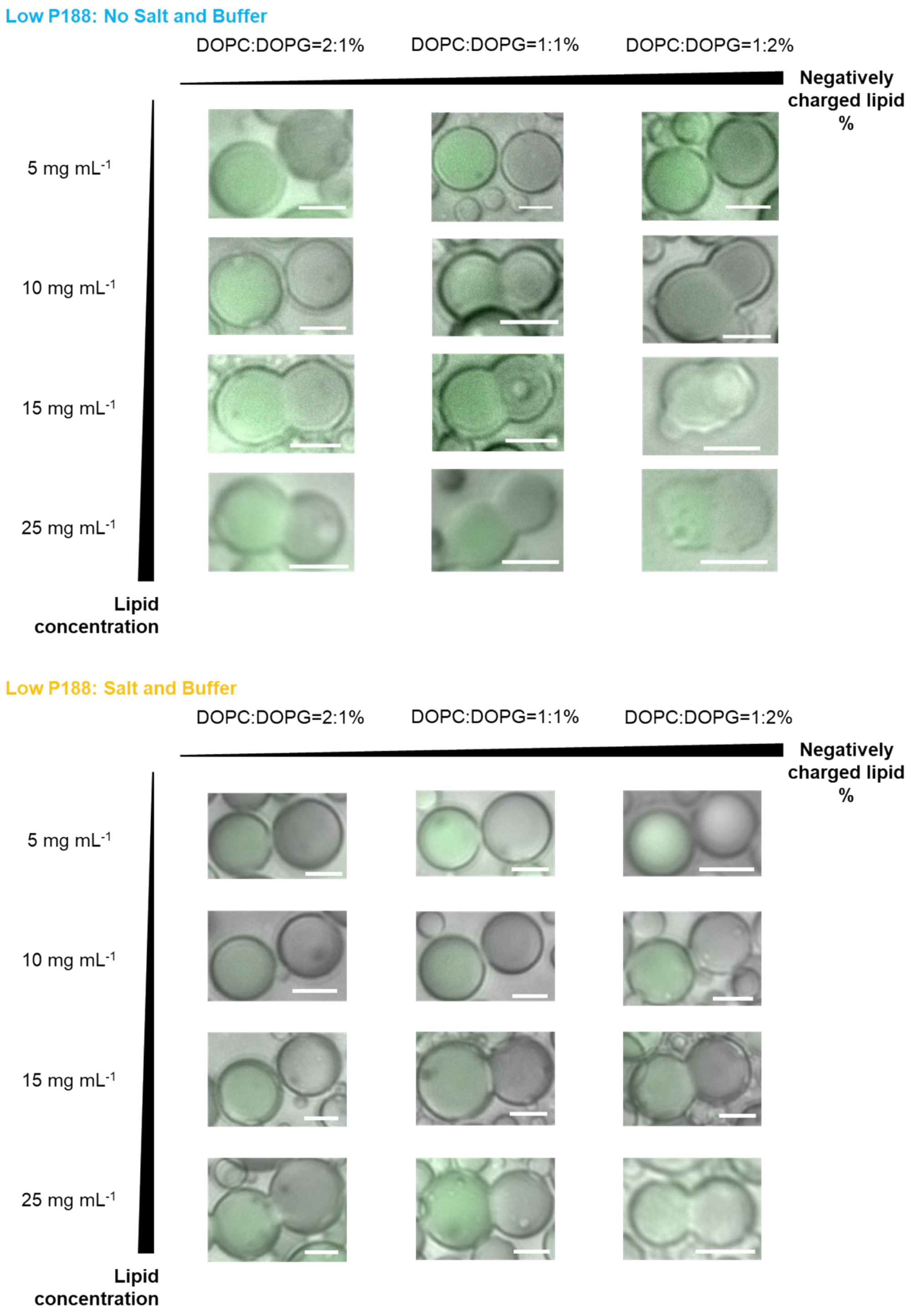

Supplementary Fig. S16 Representative brightfield images, with colocalized fluorescence, of droplet adhesion in the low P188 regime. Matrices show the effect of lipid concentration and negative charge (DOPC:DOPG ratios) without (top) and with (bottom) salt and buffer. Scale bar: 10 µm.

Supplementary Tables

P188 Concentration in OA	On-chip Dewetting Time
0.1%	2.9 (±0.3) sec
5%	30.8 (±2.2) sec

Supplementary Table S1 On-chip dewetting times for 0.1% and 5% P188 in the OA phase (no salt). Conditions: 25 mg/mL 1:2 DOPC:DOPG, 1.2 confinement, and 1.2 mm/s flow speed.

A

PC:PG	mg/mL	γ_{12}	γ_{23}
2:1	5	1.76	1.36
	10	1.15	0.78
	15	0.79	0.31
1:1	5	1.36	0.68
	10	0.49	N.A.
	15	N.A.	N.A.
1:2	5	0.98	0.46
	10	0.34	N.A.
	15	N.A.	N.A.

B

PC:PG	mg/mL	γ_{12}	γ_{23}
2:1	5	1.76	N.A.
	10	1.15	N.A.
	15	0.79	N.A.
1:1	5	1.36	N.A.
	10	0.49	N.A.
	15	N.A.	N.A.
1:2	5	0.98	N.A.
	10	0.34	N.A.
	15	N.A.	N.A.

Supplementary Table S2 Interfacial tension measurements (mN/m) between oil and aqueous phases obtained via pendant drop tensiometer for varied lipid compositions and P188 concentrations with salt and buffer. Values represent the surface tension between the inner aqueous (IA) and LO phases (γ_{12}) and between the LO and OA phases (γ_{23}). Conditions where the surface tension was below the measurable threshold of 0.3 mN/m are indicated as "N.A.". For both datasets, the IA solution consisted of 500 mM sucrose and 0.02% P188 at pH 7.6. The OA solution was 500 mM glucose at pH 7.6 either with (A) 0.1% P188 or (B) 5% P188.

A

PC:PG	mg/mL	γ_{12}	γ_{23}
2:1	5	2.98	1.49
	10	1.80	1.32
	15	1.47	0.94
1:1	5	2.1	1.21
	10	1.34	0.71
	15	1.21	0.53
1:2	5	1.60	0.74
	10	1.12	0.59
	15	1.01	0.38

B

PC:PG	mg/mL	γ_{12}	γ_{23}
2:1	5	2.98	0.64
	10	1.80	0.24
	15	1.47	N.A.
1:1	5	2.1	0.35
	10	1.34	N.A.
	15	1.21	N.A.
1:2	5	1.60	N.A.
	10	1.12	N.A.
	15	1.01	N.A.

Supplementary Table S3 Interfacial tension measurements (mN/m) between oil and aqueous phases obtained via pendant drop tensiometer for varied lipid compositions and P188 concentrations without salt or buffer. Values represent the surface tension between the inner aqueous (IA) and LO phases (γ_{12}) and between the LO and OA phases (γ_{23}). Conditions where the surface tension was below the measurable threshold of 0.3 mN/m are indicated as "N.A.". For both datasets, the IA solution consisted of 10 mM HEPES, 100 mM NaCl, 290 mM sucrose, and 0.02% P188 at pH 7.6. The OA solution was 10 mM HEPES, 100 mM NaCl, 290 mM glucose at pH 7.6 either with (A) 0.1% P188 or (B) 5% P188.

Supplementary References

1 S. Torza and S. G. Mason, *Science*, 1969, **163**, 813–814.
2 N.-N. Deng, W. Wang, X.-J. Ju, R. Xie, D. A. Weitz and L.-Y. Chu, *Lab Chip*, 2013, **13**, 4047–4052.
3 N.-N. Deng, M. Yelleswarapu and W. T. S. Huck, *J. Am. Chem. Soc.*, 2016, **138**, 7584–7591.
4 K. Ben M'barek, D. Ajjaji, A. Chorlay, S. Vanni, L. Forêt and A. R. Thiam, *Developmental Cell*, 2017, **41**, 591-604.e7.
5 E. Atefi, J. A. Mann and H. Tavana, *Langmuir*, 2014, **30**, 9691–9699.
6 M. Mastiani, N. Firoozi, N. Petrozzi, S. Seo and M. Kim, *Sci Rep*, 2019, **9**, 15561.
7 P. Bahadur, P. Li, M. Almgren and W. Brown, *Langmuir*, 1992, **8**, 1903–1907.
8 S. Asfia, R. Seemann and J.-B. Fleury, *Biochimica et Biophysica Acta (BBA) - Biomembranes*, 2023, **1865**, 184074.
9 M. Schaich, D. Sobota, H. Sleath, J. Cama and U. F. Keyser, *Biochimica et Biophysica Acta (BBA) - Biomembranes*, 2020, **1862**, 183359.
